# Floats with bio-optical sensors reveal what processes trigger the North Atlantic bloom

**DOI:** 10.1038/s41467-017-02143-6

**Published:** 2018-01-15

**Authors:** A. Mignot, R. Ferrari, H. Claustre

**Affiliations:** 1 Massachusetts Institute of Technology, Cambridge, MA 02139 USA; 2 Laboratoire d’Océanographie de Villefranche (LOV), UPMC Univ. Paris 06, CNRS, UMR 7093, Sorbonne Universités, 181 Chemin du Lazaret, 06230 Villefranche-sur-mer, France

## Abstract

The North Atlantic bloom corresponds to a strong seasonal increase in phytoplankton that produces organic carbon through photosynthesis. It is still debated what physical and biological conditions trigger the bloom, because comprehensive time series of the vertical distribution of phytoplankton biomass are lacking. Vertical profiles from nine floats that sampled the waters of the North Atlantic every few days for a couple of years reveal that phytoplankton populations start growing in early winter at very weak rates. A proper bloom with rapidly accelerating population growth rates instead starts only in spring when atmospheric cooling subsides and the mixed layer rapidly shoals. While the weak accumulation of phytoplankton in winter is crucial to maintaining a viable population, the spring bloom dominates the overall seasonal production of organic carbon.

## Abstract (web summary)

The drivers of North Atlantic phytoplankton bloom have been debated for decades, partially owing to incomplete sub-surface observations. Here, Mignot et al. use robotic sensors to provide detailed observations of developing blooms and to explore the drivers of different phases of plankton growth.

## Introduction

The bloom in the sub-polar North Atlantic represents the most dramatic seasonal increase in phytoplankton biomass for the global ocean. The organic carbon synthesized during the bloom is believed to provide the bulk of the energy required to support the entire marine food chain of the area and contributes significantly to global ocean CO_2_ uptake^1^. The event is referred to as the North Atlantic spring bloom, because in situ^2^ and satellite observations^3,4^ show that this dramatic biomass increase occurs in spring. Recent work^5^ has provided evidence that some growth of phytoplankton populations can be observed as early as the beginning of winter, but at concentrations too low to be easily detected. This result has raised the question of whether this winter population growth represents the beginning of the bloom (“a rapid excessive growth of plankton population” as per the William and Webster dictionary definition) or a period of weak population growth preceding the more explosive growth later in the season which characterizes the proper bloom.

The textbook explanation for what triggers the North Atlantic bloom stems from the seminal works of Gran and Braarud^6^, Riley^7^, and Sverdrup^8^. A phytoplankton bloom develops when the phytoplankton division rates within a given population (*µ*_p_, where the subscript p stands for population), rapidly exceed the loss rates (*l*_p_) from grazing, viral lysis, etc., such that the net population growth (or biomass accumulation) rate, *r*_p_ = *µ*_p_ − *l*_p_, becomes positive. The argument goes that, in the North Atlantic, the bloom begins in spring when *µ*_p_ rapidly increases and exceeds *l*_p_ in response to a reduction in the vertical extent^3,4,8^ or in the strength^9,10^ of turbulent mixing in the upper ocean—it is the strong mixing that suppresses cell division by keeping phytoplankton cells away from the well-lit surface during winter. The spring decrease in turbulent mixing is very rapid and thus, in this view, the division rates are expected to increase very rapidly, while changes in loss rates lag behind until the consumers catch up with the growing phytoplankton population. Consequently, the onset of the bloom is predicted to result in a rapid increase in *µ*_p_ and *r*_p_ with comparatively little change in *l*_p_.

This paradigm has been recently challenged. Using satellite observations of phytoplankton concentrations and mixed layer depth estimates from climatology to extrapolate the vertical integral of phytoplankton from the surface concentrations, Behrenfeld^5^ reported that a slow accumulation of phytoplankton standing stocks begins in early winter, when mixing is still deep and vigorous. He further pointed out that this early stock accumulation was missed in previous analysis of surface concentrations from satellite, because the deepening of the mixing layer in winter diluted the surface concentrations even though the vertical integral was increasing. In order to explain this winter phytoplankton biomass accumulation, Behrenfeld argued that the net population growth was triggered by a decrease in loss rates rather than an increase in division rates, which were still close to their minimum in early winter. The loss rates supposedly dropped because the phytoplankton-zooplankton encounter rates decrease in winter, when mixing penetrates deeper in the water column and dilutes both populations. Behrenfeld’s observations further suggested that no explosive growth in phytoplankton populations (i.e., rapid increase in *r*_p_) developed in spring. In order to explain the lack of acceleration in spring population growth, it was presumed that any increase in phytoplankton cell division was matched by a corresponding increase in grazing rates. According to these observations, the bloom appeared to start in winter and it developed over many months with low rates of accumulation and without any acceleration in spring.

Determining which paradigm best describes the typical phytoplankton phenology in late winter in the North Atlantic has proven very challenging, because detailed high-frequency observations of the vertical profile of phytoplankton biomass from autumn to spring are quite rare. Ships can only sample limited regions for a few weeks, while the bloom is very heterogeneous in space and its development spans months. Satellite remote sensing of ocean color measures only the surface phytoplankton concentration and returns incomplete maps due to cloud cover, especially at high latitudes. These limitations have only recently been overcome through the development of miniaturized bio-optical sensors installed on profiling floats, the so-called Biogeochemical-Argo (BGC-Argo) floats, which acquire time series of key variables whatever the sea conditions and over several full annual cycles^11^.

In this study, we use BGC-Argo float profiles from the sub-polar North Atlantic to quantify the net population growth rates in winter and spring and provide a detailed description of the typical evolution of phytoplankton during these seasons. A major advance over previous studies is that we rely on multiple floats and many years, so that we can derive robust conclusions about what is the typical phenology of phytoplankton in winter and spring. Our main conclusion is that phytoplankton populations start increasing in winter, but at very weak rates, while the explosive acceleration in these rates, typical of blooms, is not observed until spring.

## Results

### Data sources and processing

Our study is based on data collected by nine autonomous BGC-Argo floats that profiled in the sub-polar North Atlantic from the surface to 1000 m, every 1–10 days (Supplementary Table 1). The BGC-Argo float data were downloaded from the Argo Global Data Assembly Centre (Argo GDAC) in France^12^. The floats were deployed in 2013 and they returned nine time series of the 2013–2014 bloom, seven time series of the 2014–2015 bloom and five time series of the 2015–2016 bloom. For each yearly time series, we focus on the period from September to August to fully cover the seasons of interest, winter through spring.

The BGC-Argo floats were instrumented with miniaturized CTD and bio-optical sensors and measured vertical profiles of temperature, salinity, pressure, chlorophyll *a* fluorescence (*Chl*, in mgChl m^−3^), particle backscattering coefficient at 700 nm (*b*_bp_, in m^−1^), and instantaneous photosynthetically available radiation (*iPAR*, in μmol photons m^−2^ s^−1^). Temperature and salinity were used to compute the potential density of sea water and determine the depth of the mixed layer where potential density is well homogenized (as a proxy for the layer where mixing is active). Following de Boyer Montégut et al.^13^, the mixed layer depth (*H* in m) was computed as the depth at which the change in potential density from its value at 10 m, *∆σ*_*θ*_, exceeded 0.03 kg m^−3^— this value of *∆σ*_*θ*_ best tracked the region of weak stratification in our data set. The euphotic layer depth (*H*_e_ in m), the depth below which the light level is too low to support photosynthesis, was computed as the depth at which the daily-averaged photosynthetically available radiation drops below 0.1 mol photons m^−2^ d^−1^, corresponding to the lowest light levels at which the temperate diatom *Phaeodactylum tricornutum* has been observed to grow^14^. (Diatoms are expected to dominate the phytoplankton population during the North Atlantic bloom^2,15^.) Other criteria to define mixed layer and euphotic layer depths can be found in the literature and they all have limitations. In the Supplementary Information, we show that our results are quite insensitive to the specific criteria used (Supplementary Note 1). Phytoplankton carbon biomass (*P* in mgC m^−3^) was estimated from *b*_bp_ as explained in the Methods section. Finally, the daily mean and daily maximum (the value recorded at 15 h GMT, close to local noon) heat fluxes along the float trajectories were generated by extracting from the ECMWF ERA-interim reanalysis^16^ hourly net atmospheric heat fluxes (in W m^−2^) the pixel value closest to the float daily positions. We ignored the freshwater fluxes as well as winds that are minor contributors to winter vertical mixing in the North Atlantic^17^.

**Fig. 1:**
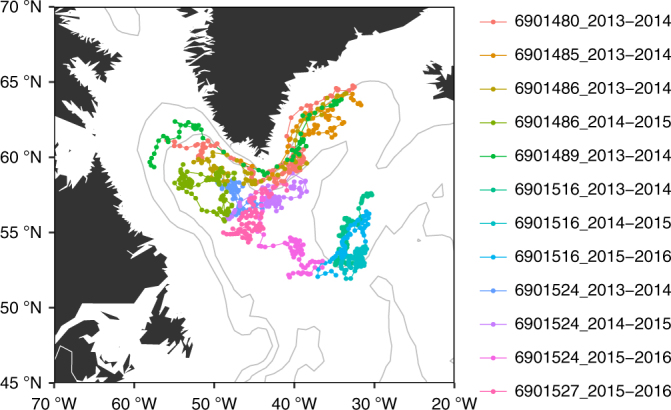
Locations of the 12 time series analysed in the study. The name of the various time series includes the float’s World Meteorological Organization (WMO) number followed by the 2 years sampled by the floats. Note that a same float can acquire several time series

In 2 out of the 21 time series sampled by the floats, the winter-spring temporal variations in phytoplankton biomass were accompanied by significant changes in both temperature and salinity. The concurrent changes in biological and physical variables suggest that these variations were most likely the result of floats crossing a water mass boundary and reflected spatial variations in biomass rather than local population growth. In another seven time series, transient restratification was observed while the air–sea buoyancy fluxes were still negative. This can only happen if the mixing generated by surface cooling was suppressed by lateral advection of a nearby lighter water mass or by slumping of lateral density fronts through instabilities^18,19^ resulting in restratification and shoaling of the mixed layer. In practice, we assumed that lateral processes were important if restratification started before the ECMWF ERA-interim reanalysis^16^ detected any heating at the float locations during the day, after verifying that the North Atlantic air-sea buoyancy fluxes in winter and early spring were dominated by the air–sea heat fluxes. The present analysis thus focuses on the remaining 12 time series, which sampled the ocean in regions of weak water mass contrasts during winter and spring. For these floats, we could safely assume that the bloom dynamics was quasi-one-dimensional and well described by one-dimensional vertical profiles. These 12 time series were located between 50–65° N and 30–60° W (Fig. 1) and they are representative of two of the largest biogeographical provinces in the sub-polar North Atlantic^20^ covering ~60% of the area.

### Phenology of phytoplankton from winter to spring

**Fig. 2:**
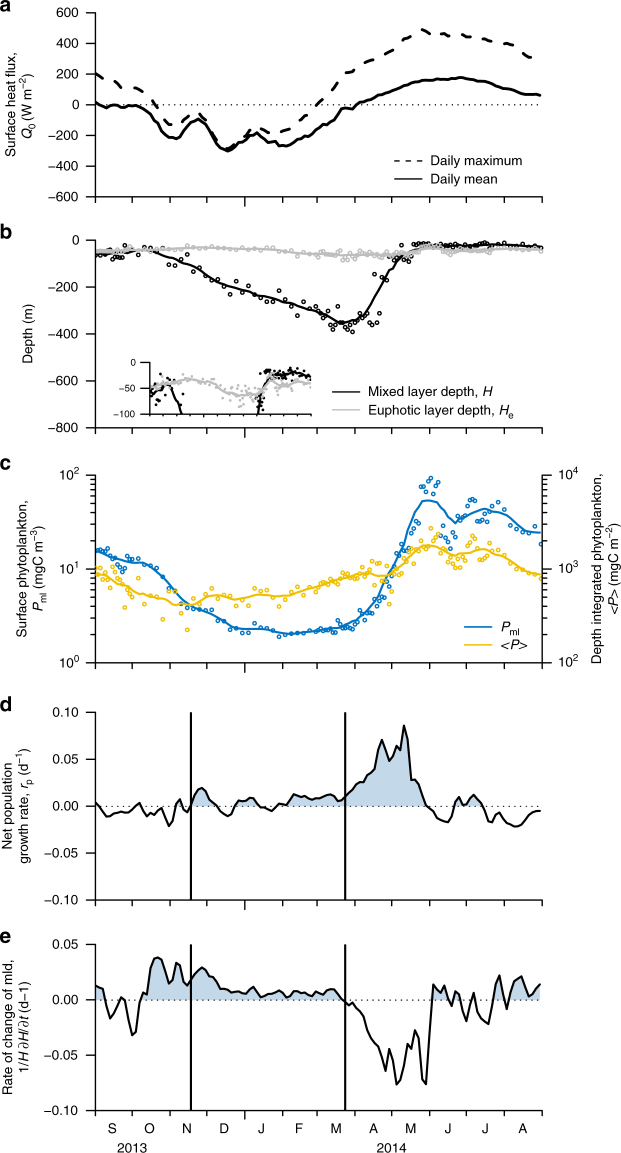
Time series of key variables measured or derived from float 6901516 from September 2013 to July 2014. **a** Daily average surface heat flux (black continuous lines) and the maximum surface heat flux at 15 h GMT, close to the local noon (black dashed lines). **b** Mixed layer (*H*, black circles) and euphotic layer depths (*H*_e_, gray circles). A zoomed view of *H* and *H*_e_ for depths shallower than 100 m is included as an inset. **c** Mixed layer averaged phytoplankton carbon biomass concentration (*P*_ml_, blue circles), and depth-integrated phytoplankton carbon biomass (${\langle P \rangle}$, yellow circles). The continuous lines in panels **a**–**c** represent 24-day running averages that remove short-term fluctuations. **d** Net population growth rate, *r*_p_. **e** Rate of change of mixed layer depth, $\frac{1}{H}\frac{\partial H}{\partial t}$, computed from the 24-day average mixed layer depth. The first vertical line marks the initiation of the weak winter accumulation phase, computed as the time when *r*_p_ becomes positive for at least 24 days. The second vertical line marks the initiation of the spring bloom, computed as the time when $\frac{1}{H}\frac{\partial H}{\partial t}$ becomes negative for at least 24 days

The physical and biological variables underwent the same qualitative cycle from winter to spring in all 12 time series. An illustration is given in Fig. 2 (all 12 time series are displayed in Supplementary Figs. 1–12). The winter–spring evolution of phytoplankton biomass is characterized by two distinct phases. The first phase begins shortly after the mixed layer starts deepening in late fall/early winter in response to surface cooling. During this phase, the depth-integrated phytoplankton carbon biomass (${\langle P \rangle}$ in mgC m^−2^; computed down to the base of the mixed layer or the euphotic layer, whichever is deeper) starts increasing. The mixed-layer-averaged phytoplankton carbon biomass concentration (*P*_ml_ in mgC m^−3^) instead continues to slowly decrease because the increase in vertically integrated phytoplankton is offset by its dilution throughout a progressively deeper mixed layer. The second phase starts in early spring when the mixed layer suddenly shoals and *P*_ml_ increases very rapidly. The increase in ${\langle P \rangle}$ is less rapid than that in *P*_ml_ due to detrainment of phytoplankton through the shoaling mixed layer base. Figure 2a further shows that the shoaling in mixed layer depth occurs some time after the first seasonal detection of heating during the day and just before the time when the daily-averaged heat flux turns positive. This is consistent with the hypothesis that the end of wintertime convection triggers the increase in surface phytoplankton concentration, as surmised by Taylor and Ferrari^10^, but it remains unclear how many hours of daily heating are necessary for restratification to start.

The phenology of phytoplankton populations is best quantified in terms of the rate of net population growth, *r*_p_, and the rate of change of mixed layer depth, $\frac{1}{H}\frac{\partial H}{\partial t}$. The rate of change of mixed layer depth represents the rate of dilution/detrainment of biomass: dilution, if positive, and detrainment, if negative. *r*_p_ is a measure of how fast the overall population grows or decays.

When the mixed layer is deeper than the euphotic layer, *r*_p_ is computed as $\frac{1}{{ \langle P\rangle}}\frac{\partial \langle P\rangle}{\partial t}$ when the mixed layer is deepening and $\frac{1}{{P_{{\mathrm{ml}}}}}\frac{\partial P_{ml}}{\partial t}$ when the mixed layer is shoaling. When the mixed layer is shallower than the euphotic layer, *r*_p_ is computed as $\frac{1}{{ \langle P\rangle}}\frac{\partial \langle P\rangle}{\partial t}$ regardless of whether the mixed layer is shoaling or not. As explained in the Methods section, this guarantees that *r*_p_ captures the balance between division and loss rates of the phytoplankton population and not the dilution/detrainment of biomass due to mixed layer deepening/shoaling. For completeness, we also computed *r*_p_ using *Chl* instead of phytoplankton carbon biomass *P* from *b*_bp_ and found very similar results as verified in many other studies in the North Atlantic^5,21,22^ (Supplementary Note 2). Similarly, we derived mixed-layer-averaged chlorophyll *a* concentrations from radiometric measurements and found very similar patterns (Supplementary Note 3). In order to compute *r*_p_ and $\frac{1}{H}\frac{\partial H}{\partial t}$, the time series of *P*_ml_, ${\langle P \rangle}$, *H*, and *H*_e_ were linearly interpolated on equally spaced 3-day time series, the average sampling period in our dataset. Then, a running average of 24-days was applied to the time series, so as to filter out short-term fluctuations and focus on the seasonal evolution of phytoplankton biomass. (The 24 days correspond to the spring bloom e-folding timescale as shown in Supplementary Note 4.)

The time series of *r*_p_ and $\frac{1}{H}\frac{\partial H}{\partial t}$ in Fig. 2d, e confirm that growth of phytoplankton populations begins in winter at a time when the mixed layer is still deepening ($\frac{1}{H}\frac{\partial H}{\partial t} > 0$). The net population growth rates are positive, but weak of order of 0.01 d^−1^. During this phase of weak biomass accumulation, *r*_p_ is of the same order as $\frac{1}{H}\frac{\partial H}{\partial t}$ and the phytoplankton concentration *P*_ml_ decreases only slightly, because the net population growth and dilution approximately balance. Growth can only be seen in the total integrated biomass, ${\langle P \rangle}$, which is not affected by dilution. A sudden acceleration in the net population growth rate is seen when $\frac{1}{H}\frac{\partial H}{\partial t}$ changes sign, i.e. when there is a shift from atmospheric cooling to heating, at least for a few hours during the day, and the mixed layer starts shoaling. This acceleration represents the onset of the spring bloom. Hereinafter we will refer to the winter phase of weak biomass accumulation as the “weak winter accumulation phase” to distinguish it from the following explosive “spring bloom” phase.

**Fig. 3:**
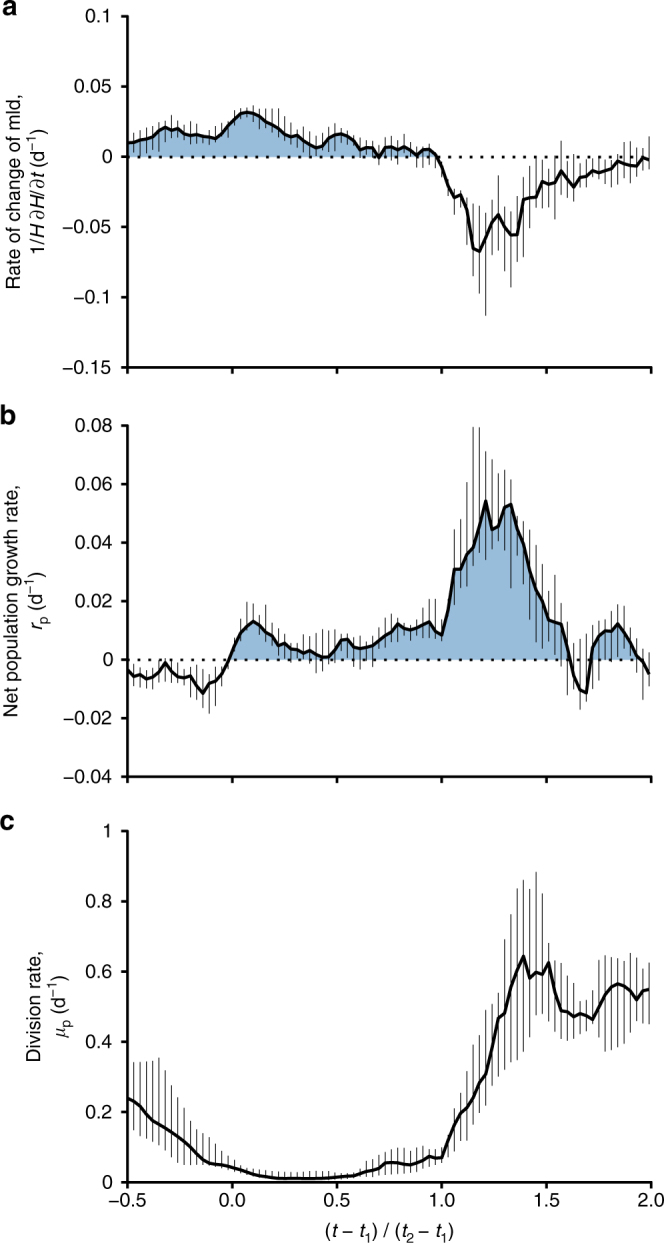
Rate of change of physical and biological variables. Median (solid thick line) and interquartile range (vertical bars) of the rate of change of mixed layer depth (**a**, $\frac{1}{H}\frac{\partial H}{\partial t}$), the net phytoplankton population growth rate (**b**, *r*_p_), and phytoplankton division rates (**c**, *μ*_p_), estimated from the 12 time series collected by the floats. The time axis is rescaled by the onset times of the weak winter accumulation phase (*t*_1_) and the spring bloom (*t*_2_) introducing $\tau = \left( {t - t_1} \right)/\left( {t_2 - t_1} \right)$. *τ* = 0 corresponds to the initiation of the weak winter accumulation phase and *τ* = 1 corresponds to the initiation of the spring bloom. The time span between *τ* = 0 and *τ* = 1 corresponds to the duration of the weak winter accumulation phase and it is ~120 days long

The same phenology can be observed by sifting through all the float data, available in the Supplementary Information. Alternatively, we derive a method to combine all 12 time series into a unified picture. The main challenge to averaging all float data is that the timing of the two phases is highly variable from year to year. The time axis for time series is therefore rescaled by the onset times of the weak winter accumulation phase (*t*_1_) and the spring bloom (*t*_2_). The onset time of the weak winter accumulation phase is computed as the first time in winter when *r*_p_ becomes positive for at least 24 days—the 24 days criterion is imposed to be consistent in ignoring high frequency fluctuations. The onset of the spring bloom is computed as the time when the mixed layer starts to restratify, i.e., when $\frac{1}{H}\frac{\partial H}{\partial t}$ becomes negative for at least 24 days. The new time axis is then defined as $\tau = \left( {t - t_1} \right)/\left( {t_2 - t_1} \right)$. *τ* = 0 corresponds to the initiation of the weak winter accumulation phase and *τ* = 1 corresponds to the initiation of the spring bloom. The time span between *τ* = 0 and *τ* = 1 corresponds to the duration of the weak winter accumulation phase and it lasts for ~ 120 days. Figure 3a, b show the median and the interquartile range of $\frac{1}{H}\frac{\partial H}{\partial t}$ and *r*_p_ over all 12 time series as a function of *τ*. In all time series, *r*_p_ becomes positive, marking the onset of the weak winter accumulation phase, when the mixed layer is still deepening. During this phase, the net population growth rates are weak and never exceeds 0.02 d^−1^, corresponding to a population doubling time of more than a month. The bloom begins in spring, as soon as the mixed layer starts shoaling, with a sudden spike in net population growth rate, that reaches values as high as 0.08 d^−1^, corresponding to a net population doubling time of 9 days. The 12 time series therefore confirm that the weak winter accumulation phase develops while the mixed layer is deepening, while the spring bloom is associated with a decrease in vertical mixing and the associated shoaling of the mixed layer.

The analysis has so far established a correlation between changes in mixed layer depth and bloom phenology. In order to test the hypothesis that the changes in mixed layer depth cause the changes in net population growth rates, we need estimates of the overall cell division rates *μ*_p_—the net population growth rates are equal to the difference between division and loss rates. While *μ*_p_ cannot be measured directly with autonomous platforms, a phytoplankton growth model^23^ can be used to estimate *μ*(*z*, *t*) at depth *z* and time *t*, based on the float profiles of temperature, *iPAR*, *Chl*, and *P* and two additional photosynthesis parameters^24^ that characterize the population present in the water column (see Methods). The daily-averaged primary production is then computed as $PP = \frac{1}{{1{\mathrm{day}}}}\mathop {\int }\nolimits_0^{1{\mathrm{day}}} \mu \left( {z,t} \right)P(z){\mathrm{d}}t$ and the division rates for the overall population is derived as $\mu _{\mathrm{p}} = \langle PP\rangle/\langle P\rangle$, where the angle brackets here denote an integration down to the base of the mixed layer or the euphotic layer, whichever is deeper. These semi-analytical models of phytoplankton growth carry substantial uncertainties due to errors in both the estimates of the input variables and the photosynthesis parameters^25,26^. We show in the Supplementary Information that these uncertainties affect primarily the magnitude of *μ*_p_ (and similarly ${\langle PP \rangle}$), but not as much its temporal evolution (Supplementary Note 5). Consequently, we will focus on the seasonal variations of *μ*_p_, but we will not attempt any comparison of the absolute values of *μ*_p_ and *r*_p_. The median and the interquartile range of *μ*_p_ over all 12 time series are shown in Fig. 3c; the times series of *μ*_p_ for each float were smoothened with a 24-day running average to be consistent with the other time series. Phytoplankton division rates are still decreasing at *τ* = 0, when the weak winter accumulation phase starts, because the light experienced by the overall population diminishes in response to the deepening mixed layers and the declining incoming solar radiation. The division rates begin to increase only half way through the weak winter accumulation phase, when the sea surface light starts increasing. However, *μ*_p_ does not increase much, because the strong atmospheric cooling continuously mixes phytoplankton out of the euphotic layer. Division rates peak up only when the mixing subsides at the end of winter and the cells remain near the surface where light is abundant.

The phytoplankton growth model reveals that the weak winter accumulation phase starts when division rates *μ*_p_ are still declining. In order for *r*_p_*=μ*_p_ − *l*_p_ to turn positive while *μ*_p_ is still declining, the loss rates *l*_p_ must be decreasing even faster than *μ*_p_. While a decrease in *l*_p_ is consistent with Behrenfeld’s scenario, we cannot prove that the decrease is caused by a deepening of the mixed layer and hence a reduction in phytoplankton–zooplankton encounter rates and the associated grazing rates. Such a proof would require accurate estimates of *μ*_p_ − *r*_p_, which is beyond our reach due to the large uncertainties in the phytoplankton growth model. The model however clearly shows the dramatic increase in division at the beginning of the spring bloom, when convection in the upper ocean stops. The concomitant increase in net population growth rate further suggests that, at the onset of the spring bloom, phytoplankton grow somewhat unchecked by their consumers, consistent with Sverdrup’s paradigm.

**Fig. 4:**
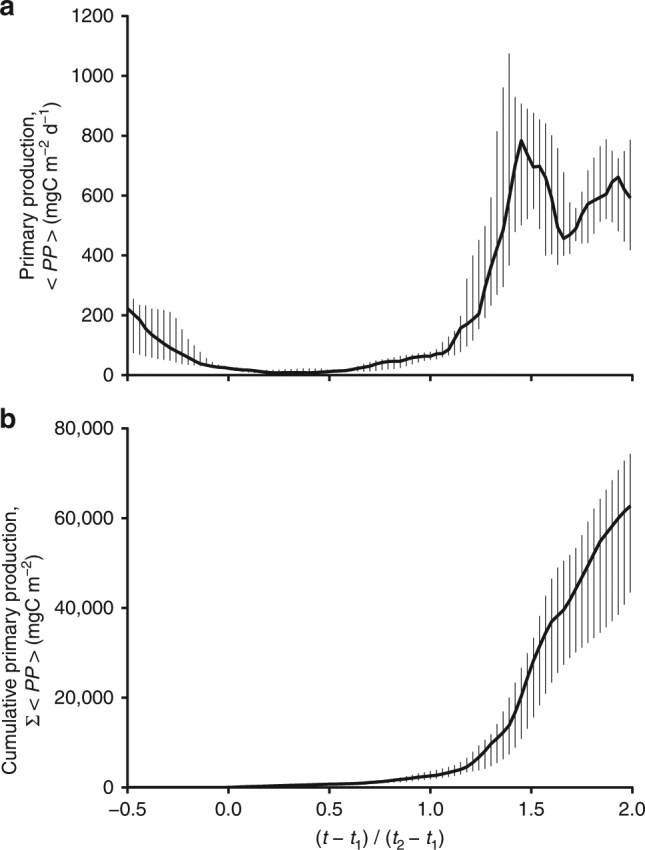
Phenology of primary production. Same as Fig. 3, but for the daily averaged and depth-integrated primary production, ${\langle PP \rangle}$ (**a**) and the cumulative temporal integral of ${\langle PP \rangle}$ starting at τ = 0, ${\Sigma \langle PP \rangle}$ (**b**)

It is instructive to compare the amount of organic carbon produced during the weak winter accumulation phase and during the spring bloom. Daily-averaged and depth-integrated primary production, ${\langle PP \rangle}$ were computed using the phytoplankton growth model described above. Figure 4a shows the median and the interquartile range of ${\langle PP \rangle}$ over all 12 time series. ${\langle PP \rangle}$ is low (<50 mgC m^−2^ d^−1^) during the weak winter accumulation phase, because strong mixing keeps cells away from the well-lit surface ocean. When vertical mixing weakens in spring, ${\langle PP \rangle}$ explodes and reaches maximum values as high as 900 mgC m^−2^ d^−1^, because of the combined effect of large concentration of phytoplankton in the euphotic layer and the outburst in cell division rates. We conclude that the production of organic carbon is dominated by the spring bloom as evident in Fig. 4b, which shows the cumulative temporal integral of the ${\langle PP \rangle}$ time series starting at *τ* = 0.

## Discussion

There is an ongoing debate as to whether the North Atlantic bloom starts in response to a spring shoaling of the mixed layer and the associated decrease in mixing, as surmised by Sverdrup^8^, or in response to a winter deepening in the mixed layer and the associated decrease in grazing rates, as suggested by Behrenfeld^5^. An analysis of 12 yearly time series collected by BGC-Argo floats suggests that accumulation of phytoplankton biomass starts in early winter, when the mixed layers are still deepening, in response to intense air–sea cooling, and light levels are decreasing. However, the net population growth rates and the primary production remain very weak throughout this phase. The bloom starts in earnest when net population growth rates and production of organic carbon explode at the beginning of spring in response to a shift from atmospheric cooling to heating, at least for a few hours during the day, which drives a rapid shoaling of the mixed layer.

 For completeness we carried out the same analysis on the seven time series perturbed by a winter transient restratification, which were excluded from the presentation so far (Supplementary Fig. 13). Overall, the same phenology is observed: a weak winter accumulation phase followed by a spring bloom. However, in these seven time series the temporal variability was higher than in the 12 quasi-one-dimensional time series; fluctuations in mixed layer depth in winter triggered occasional short-lived blooms and the springtime restratification occasionally preceded the shutdown of local sea surface cooling.

It is unclear whether the weak winter accumulation phase is a necessary precursor to the spring bloom. The hypothesis could be rejected if a float sampled a spring bloom without a preceding weak winter population growth, but this was not observed in any of the 12 time series. Boss and Behrenfeld^21^ analysed the development of blooms from a float released a bit further south than the floats analysed here. Their float stayed in the water for 2 years. In the second year, the phenology was consistent with our findings: a weak population growth started in winter and a rapid acceleration in population growth rates followed in spring. In the first year, instead, the weak winter accumulation phase was very short or altogether missing. It is possible that this time series represent an example of a spring bloom without a weak winter accumulation phase. However, caution should be applied in interpreting data from this float, because the temperature and salinity fields changed substantially from winter to spring^27^. This raises the possibility that lateral variations in the environment rather than biological processes affected the evolution of biomass.

Our results beg the question of whether the weak winter accumulation phase requires deep mixed layers typical of the high latitude open ocean. This could be addressed by studying blooms in coastal areas and lakes where mixed layer variations are less extreme and phytoplankton dilution may be expected to play a lesser role, possibly resulting in no net population growth in winter.

Finally, we surmise that the debate on what drives the onset of the North Atlantic spring bloom largely stems from limitations in the measurement techniques. For decades, satellite ocean color has been the primary tool to investigate the controls on the initiation of the North Atlantic spring bloom, because it provides the only global and synoptic view of phytoplankton concentrations. However, satellites sample the concentrations at the ocean surface and winter images are scarce due to ubiquitous cloud coverage. As a result, satellite-based studies typically missed the weak winter accumulation phase and focused instead on the sudden spring increase in surface phytoplankton concentrations. Many studies defined the onset of the bloom as the first exceedance of a concentration threshold, thereby missing any possible winter population growth with weak surface concentrations. This literature, not surprisingly, concluded that satellite data supported Sverdrup’s hypothesis. More recently Behrenfeld showed that weak net population growth rates can be detected, albeit indirectly, from winter imagery by (1) averaging satellite phytoplankton concentrations over large areas to overcome limited winter retrievals and (2) using mixed layer depth estimates from climatology to extrapolate the vertical integral of phytoplankton from the surface concentrations. This approach provided support for weak winter population growth  but the averaging over large areas and uncertainties in the mixed layer depth estimates smeared the subsequent bloom evolution, which is very heterogeneous from place to place, and thus masked the rapid spring increase in population growth.

It is intriguing to speculate about the implication of our analysis for the response of the North Atlantic spring bloom to climate change. It is expected that in a warming climate the stratification of the upper ocean will increase^28,29^. The rate of deepening of a mixed layer is inversely proportional to the upper ocean stratification^30^ and may thus be expected to decrease. The float data show that the weak winter accumulation phase is associated with the rapid deepening of the winter mixed layer. Should the deepening slow down, it may reduce the dilution of phytoplankton and grazers and suppress the weak winter accumulation phase. Furthermore in a warming climate, the shift from cooling to warming of the surface ocean may be expected to occur earlier in the season resulting in earlier blooms, as suggested for lakes of the temperate zone^31^.

## Methods

### Float data processing

All floats used in this study were PROVOR CTS-4 profiling floats, equipped with a SEABIRD SBE41 CPCTD sensor, a Satlantic OC4 radiometer measuring downwelling irradiance at 380, 412, and 490 nm and Photosynthetically Available Radiation integrated between 400 and 700 nm, a WET Labs ECO-triplet fluorometer comprising a chlorophyll *a* fluorometer, a CDOM fluorometer and a backscattering sensor at 700 nm. The CDOM data were not used in this study. The 6901485-86 floats also included an AANDERAA optode oxygen sensor and the 6901485 float was equipped with a Satlantic SUNA nitrate sensor.

The floats nominal mission included CTD and optical profiles from 1000 m to the surface. The sampling resolution was 10 m from 1000 m to 250 m, 1 m from 250 m to 10 m, and 0.2 m from 10 m to the surface. The upward casts were repeated every 1, 2, 3, 5, or 10 days depending on the mission and scientific objectives. The floats typically emerged to the sea around local noon.

The CTD and trajectory data were quality-controlled using the standard Argo protocol^32^. Following the BGC-Argo procedure^33,34^, the optical raw signals (counts) were transformed into *Chl*, *b*_bp_ and *iPAR*. The data were quality-controlled following the BGC-Argo quality control manual^35^. In addition to this, all time series were visually inspected and we did not detect any sensor failure or drift over time. As suggested by Roesler et al.^36^, the *Chl* values were divided by a factor of 2 to account for a calibration systematic error in the WET Labs ECO-series fluorometers. Fluorescence quenching was corrected using the method of Xing et al.^37^ Finally, because winter phytoplankton concentrations are vanishingly low in the region considered, we verified that the winter *Chl* and *b*_bp_ measurements collected in the mixed layer were always greater than their respective sensors detection levels.

### Float estimates of phytoplankton carbon biomass

In situ measurements of *b*_bp_ reflect the light backscattering from a complex mixture of particles that includes both phytoplankton and non-algal particles (i.e., colloid, bacteria, biogenic detritus, minerals, and bubbles)^38^. Several studies^22,39^ have suggested that the seasonal changes in *b*_bp_ tracked the seasonal changes in phytoplankton biomass, because the non-algal particles contributing to *b*_bp_ can be separated into a “background” component stable over time and a second component that covary with phytoplankton abundance. The backscattering coefficient of particle can therefore be converted into phytoplankton carbon biomass by subtracting a background value and multiplying the remaining *b*_bp_ by a conversion factor. Following this suggestion, *b*_bp_ was converted into phytoplankton carbon biomass (*P*, mgC m^−3^).

The background value was estimated at depths where phytoplankton is absent but non-algal particles are present. For each profile, a background value was computed as the minimum *b*_bp_ value measured at depths below 900 m, where no phytoplankton is expected. The float background value was then calculated as the mean of the 10 minimum values of *b*_bp_ found for all profiles from that float.

The conversion factor was set to 11,260 mgC m^−2^. This value corresponds to an average particulate organic carbon—*b*_bp_ ratio equal to 37,537 mgC m^−2^ (a value determined from mixed layer in situ measurements during the 2008 North Atlantic bloom experiment^40^), and an average phytoplankton contribution to particulate organic carbon of 30% (consistent with in situ observations from diverse ocean regions, as suggested by Behrenfeld et al.^39^).

The use of a single conversion factor does not incorporate on the one hand, changes in phytoplankton size, taxa, and internal structure and on the other hand changes in the size and composition and internal structure of non-algal particles. Similarly, the background subtraction approach does not remove small and highly scattering bubbles injected by high winds and breaking waves. Therefore, if bubbles are present, this will result in an overestimation of phytoplankton carbon biomass. However, the winter–spring evolution of net population growth rates was qualitatively similar whether we used *Chl* or *b*_bp_ (Supplementary Note 2). This consistency gave us confidence that *b*_bp_ is a reasonable proxy for phytoplankton carbon biomass in the sub-polar North Atlantic.

### Float irradiance processing

The instantaneous photosynthetically available radiation (*iPAR*) just beneath the sea surface, (${\mathrm{\it{iPAR}}}\left( {0^ - ,t} \right)$ in µmol photons m^−2^ s^−1^), was determined through

1 $${\mathrm{\it{iPAR}}}\left( {0^ - ,t} \right) = {\mathrm{\it{iPAR}}}_{{\mathrm{clear}}}\left( {0^ - ,t} \right)\frac{{{\mathrm{\it{iPAR}}}\left( {0^ - ,t = {\mathrm{noon}}} \right)}}{{{\mathrm{\it{iPAR}}}_{{\mathrm{clear}}}\left( {0^ - ,t = {\mathrm{noon}}} \right)}},$$

where *iPAR* (0^−^, *t *= noon) is the float estimate of *iPAR* just beneath the sea surface at local noon, *iPAR*_clear_(0^−^, *t *= noon) is the theoretical estimation of *iPAR* just beneath the sea surface at local noon for the same location and time of the day than the measurement, and *iPAR*_clear_(0^−^, *t*) is the theoretical estimation of *iPAR* just beneath the sea surface at time *t*. The clear sky estimates of *iPAR* were derived using a solar irradiance model, SOLPOS, developed by the National Renewable Energy Laboratory^41^. The SOLPOS model calculates the direct solar radiation for a free-cloud sky and the solar position based on the date, and location on Earth. The daily-averaged sea surface photosynthetically available radiation (*PAR*(0^−^) in mol photons m^−2^ d^−1^) was obtained by averaging Eq. (1) over the length of the day.

The irradiance was assumed to decay exponentially with depth as per the Beer–Lambert Law. The exponential decay rate, i.e., the diffuse attenuation coefficient for photosynthetically available radiation, *K* (m^−1^), was derived from the float *iPAR* measurements by fitting a linear least square regression, constrained to pass through the origin, between ${\ln}(\frac{{{\mathrm{\it{iPAR}}}\left( {z,t = {\mathrm{noon}}} \right)}}{{{\mathrm{\it{iPAR}}}\left( {0^ - ,t = {\mathrm{noon}}} \right)}})$ and *z* in the upper 50 m―the average depth at which light intensity is 1% of its surface value in our dataset.

Following the Beer–Lambert law, the euphotic layer depth, *H*_e_, was estimated as the depth at which the daily-averaged photosynthetically available radiation drops below a threshold value, *PAR*_th_, here taken as 0.1 mol photons m^−2^ d^−1^ (see main text):

2 $${{H_{\rm e}}=-{\frac{1}{K}} \, {\rm ln}{\left({\frac {PAR_{\rm th}}{PAR (0^{-})}}\right)}}.$$

### Float estimates of net population growth rates

The changes in phytoplankton concentration *P*(*z*,*t*) in response to changes in irradiance, mortality, and vertical mixing can be described by a partial differential equation:

3 $$\frac{{\partial P\left( {z,t} \right)}}{{\partial t}} = \left[ {\mu \left( {z,t} \right) - l\left( {z,t} \right)} \right]P(z,t) + \frac{\partial }{{\partial z}}\left( {\kappa _T(z,t)\frac{{\partial P(z,t)}}{{\partial z}}} \right),$$

where *z* is the vertical coordinate, *t* is time, *μ* and *l* are the local phytoplankton division and loss rates (from grazing, viral lysis, etc.) at depth *z* and time *t*, and *κ*_*T*_(*z*,*t*) is the vertical eddy diffusivity. We ignore the effect of lateral advection of phytoplankton by oceanic currents. This is a reasonable assumption as long as phytoplankton concentrations are uniform in the horizontal. Consistently, we included in the analysis only floats that were observed to drift within laterally homogeneous waters as explained in the main text.

We assume that there is no phytoplankton flux throughout the surface and the base of the productive layer (*L*, the greatest of the mixed layer or the euphotic layer depths). Integrating Eq. (3) over *L* (indicated by angle brackets) in addition to averaging over a full day (indicated by an overbar) and assuming that *P* and *L* are constant throughout the day, the overall phytoplankton population changes with time according to

4 $$\mathop {\int }\nolimits_{ \!\!\!- L}^0 \frac{{\partial P}}{{\partial t}}{\mathrm{d}}z = \langle \bar \mu P\rangle - \langle\bar lP\rangle,$$

Dividing the left hand side of Eq. (4) by $\langle P \rangle= \mathop {\int }\limits_{ - L}^0 P(z){\mathrm{d}}z$ gives an expression for the net population growth rate:

5 $$r_{\mathrm{p}} = \frac{1}{\langle P \rangle}\mathop {\int }\nolimits_{ \!\!\!- L}^0 \frac{{\partial P}}{{\partial t}}{\mathrm{d}}z.$$

During most of the time period considered in this work, the productive layer is equal to the mixed layer depth (*L=H*) and Eq. (5) can be recast as an expression for *r*_p_ as a function of *H* and ${\langle P \rangle}$,

6 $$r_{\mathrm{p}} = \frac{1}{\langle P \rangle}\left( {\frac{{\partial \langle P \rangle}}{{\partial t}} - P( - H)\frac{{\partial H}}{{\partial t}}} \right),$$

where *P*(−*H*) is the *P* concentration below the mixed layer. Equation (6) can be equivalently written as a function of the averaged phytoplankton concentration in the mixed layer, by substituting ${P}$_ml_=${\langle P \rangle}$/*H*,

7 $$r_{\rm {p}} = \frac{1}{{P_{{\rm {ml}}}H}}\left( {H\frac{{\partial P_{{\rm {ml}}}}}{{\partial t}} - (P\left( { - H} \right) - P_{{\rm {ml}}})\frac{{\partial H}}{{\partial t}}} \right).$$

Equations (6) and (7) are the same equation written in terms of ${\langle P \rangle}$ and *P*_ml_.

When the mixed layer deepens, the entrainment of phytoplankton $\left( {\frac{{P( - H)}}{\langle P \rangle}\frac{{\partial H}}{{\partial t}}} \right)$ is likely small because there is little biomass at depth and thus to a good approximation,

8 $$r_{\mathrm{p}} = \frac{1}{\langle P \rangle}\frac{{\partial \langle P \rangle}}{{\partial t}}.$$

When the mixed layer shoals, the concentration of phytoplankton in the mixed layer *P*_ml_ is likely similar to that left behind just at the base of the mixed layer *P*(−*H*) and thus $\frac{{(P\left( { - H} \right) - P_{{\mathrm{ml}}})}}{{P_{{\mathrm{ml}}}H}}\frac{{\partial H}}{{\partial t}}$ is small. To a good approximation then Eq. (7) reduces to,

9 $$r_{\mathrm{p}} = \frac{1}{{P_{{\mathrm{ml}}}}}\frac{{\partial P_{{\mathrm{ml}}}}}{{\partial t}}.$$

Equations (8) and (9) are approximations of the full expressions for the net population growth rates, given equivalently by Eqs. (6) and (7), but valid in two different limits. Equation (8) is likely accurate during mixed layer deepening, while Eq. (9) is likely accurate during mixed layer shoaling. The accuracy of neglecting the entrainment term $\left( {\frac{{P( - H)}}{\langle P \rangle}\frac{{\partial H}}{{\partial t}}} \right)$ in winter, when the mixed layer deepens, is supported by the float data. The vertical profiles of chlorophyll *a* in the Supplementary Figs. 1–12 show that phytoplankton concentrations are close to zero below the base of the mixed layer when the winter mixed layer is deepening. The accuracy of neglecting the detrainment term $\frac{{(P_{{\mathrm{ml}}} - P( - H))}}{{P_{{\mathrm{ml}}}H}}\frac{{\partial H}}{{\partial t}}$ when the mixed layer shoals was tested by verifying that

10 $$\frac{{(P\left( { - H} \right) - P_{{\mathrm{ml}}})}}{{P_{{\mathrm{ml}}}H}}\frac{{\partial H}}{{\partial t}} \ll \frac{1}{{P_{{\mathrm{ml}}}}}\frac{{\partial P_{{\mathrm{ml}}}}}{{\partial t}}.$$

An expression for $\frac{1}{H}\frac{{\partial H}}{{\partial t}}$ can be obtained substituting into Eq. (7) the expression for *r*_p_ from Eq. (6) and Eq. (10) can then be equivalently rewritten as,

11 $$(P\left( { - H} \right) - P_{{\mathrm{ml}}})\left( {\frac{1}{\langle P \rangle}\frac{{\partial \langle P \rangle}}{{\partial t}} - \frac{1}{{P_{{\mathrm{ml}}}}}\frac{{\partial P_{{\mathrm{ml}}}}}{{\partial t}}} \right) \ll \frac{{\partial P_{{\mathrm{ml}}}}}{{\partial t}}.$$

Supplementary Figure 14 shows the median of the left-hand side term of Eq. (11) computed over the 12 time series is one order of magnitude smaller than the median of right-hand side term of Eq. (11) during the restratification of the mixed layer (*τ*>1), suggesting that net population growth rates can be reasonably estimated from Eq. (9) when the mixed layer is shoaling.

Finally, toward the end of the bloom, when *H*_e_ > *H* and as long as the temporal variations in *H*_e_ or the *P* concentration below the euphotic layer are small, net population growth rates are well approximated by Eq. (8) with $P = \mathop {\int }\nolimits_{ - H_{\mathrm{e}}}^0 P{\mathrm{d}}z$.

### Float estimates of population division rates

The term $\langle\bar \mu P\rangle$ in Eq. (4) represents the daily-averaged and depth-integrated primary production, ${\langle PP \rangle}$, and the division rates for the overall population can then be estimated as *μ*_p_=${\langle PP \rangle}$/${\langle P \rangle}$, where the angle brackets denote an integration over the productive layer. We use a phytoplankton growth model^23^ to estimate *μ*(*z*,*t*) at depth *z* and time *t*, based on float temperature, light, *Chl* and *P* profiles and two prescribed photosynthesis parameters from Antoine and Morel^24^. The model resolves the daily time dependence of sea surface incoming solar radiation, as per Eq. (1). However, the model assumes constant mixed layer depth, light attenuation coefficient, *Chl* and *P* over the length of the day because the floats take only one vertical profile at local noon on days when they come to the surface.

The relationship between division rate and light is represented by

12 $$\mu \left( {z,t} \right) = \frac{{Chl\left( z \right)}}{{P(z)}}P_{{\mathrm{max}}}^B\left[ {1 - e^{ - \frac{{\alpha ^B{\mathrm{\it{iPAR}}}\left( {z,t} \right)}}{{P_{{\mathrm{max}}}^B}}}} \right]$$

where $P_{{\mathrm{max}}}^B$ is the maximum chlorophyll-specific light-saturated photosynthesis (mgC mgChl^−^^1^ s^−1^), *α*^*B*^ is the chlorophyll-specific initial slope of the photosynthesis-irradiance curve (mgC mgChl^−^^1^ s^−1^/µmol photons m^−2^ s^−1^) and *iPAR*(*z*,*t*) is the instantaneous vertical profile of photosynthetically available radiation (µmol photons m^−2^ s^−1^). Following Antoine and Morel^24^, *α*^*B*^ was set to 6.4 × 10^−6^ and $P_{{\mathrm{max}}}^B$ was calculated as an exponential function of the mixed layer-averaged potential temperature (*MLT*) assuming a two-fold increase in $P_{{\mathrm{max}}}^B$ with a 10 °C increase in temperature, i.e.,

13 $$P_{{\mathrm{max}}}^B = P_{{\mathrm{max}}}^B(20^\circ ) \times 1.065^{({\mathrm{\it{MLT}}} - 20)},$$

where $P_{{\mathrm{max}}}^B(20^\circ ) = 1.3 \times 10^{ - 3}$ mgC mgChl^−^^1^ s^−1^_._ Finally, the vertical profile of *iPAR* (μmol photons m^−2^ s^−1^) was modelled through:

14 $${\mathrm{\it{iPAR}}}\left( {z,t} \right) = {\mathrm{\it{iPAR}}}\left( {0^ - ,t} \right)e^{Kz},$$

where *iPAR*(0^−^, *t*) is the *iPAR* just beneath the sea surface, in µmol photons m^−2^ s^−1^, and *K* (m^−1^), is the diffuse attenuation coefficient for photosynthetically available radiation.

Last, we checked that phytoplankton cells move fast enough up and down through the mixed layer to experience the full vertical structure of light throughout the day. This assumption is built in the phytoplankton growth model where the population is assumed to be uniformly distributed in the vertical, while the model retains the full dependence on the instantaneous and depth-dependent irradiance. In a previous paper^42^, we derived a scaling law that estimates the overturning timescale of phytoplankton cells in a mixing layer forced by surface cooling, $T = \pi H/\sqrt 2 A\left| {B_0H} \right|^{1/3},$ where *B*_0_ is the surface buoyancy flux and *A* is a coefficient of proportionality set to 0.45^43^. The average overturning timescale in winter (between *τ* = −0.5 and *τ* = 1) is 0.8 ± 0.5 days, suggesting that within a given day, all phytoplankton cells experience all light conditions in the mixed layer and winter primary production and phytoplankton division rates are reasonably captured by the phytoplankton growth model.

### Data availability

These data were collected and made freely available by the International Argo Program and the national programs that contribute to it (http://www.argo.ucsd.edu, http://argo.jcommops.org). The Argo Program is part of the Global Ocean Observing System. The BGC-Argo data used for this study can be downloaded from ftp://ftp.ifremer.fr/ifremer/argo. The data accession DOI is 10.17882/42182.

## Electronic supplementary material

Supplementary Information
