# Author Correction: Floats with bio-optical sensors reveal what processes trigger the North Atlantic bloom

**DOI:** 10.1038/s41467-018-04181-0

**Published:** 2018-05-04

**Authors:** A. Mignot, R. Ferrari, H. Claustre

**Affiliations:** 1 Massachusetts Institute of Technology, Cambridge, MA 02139 USA; 2 Laboratoire d’Océanographie de Villefranche (LOV), UPMC Univ. Paris 06, CNRS, UMR 7093, Sorbonne Universités, 181 Chemin du Lazaret, 06230 Villefranche-sur-mer, France

Correction to: *Nature Communications* 10.1038/s41467-017-02143-6, published online 15 Jan 2018.

In the original version of this Article, the data accession 10.17882/42182 was omitted from the Data Availability statement.

In the first paragraph of the Methods subsection entitled ‘Float data processing’, the WET Labs ECO-triplet fluorometer was incorrectly referred to as ‘WETLabs ECO PUK’. In the final paragraph of this subsection, the WET Labs ECO-series fluorometer was incorrectly referred to as ‘WETLabs 413 ECO-series’.

In the Methods subsection ‘Float estimates of phytoplankton carbon biomass’, the average particulate organic carbon–bbp ratio of 37,537 mgC m^−2^ was incorrectly given as 37,357 mgC m^−2^.

In the second paragraph of the Methods subsection ‘Float estimates of population division rates’, the symbol for Celsius (C) was omitted from the phrase ‘a 10°C increase in temperature’.

These errors have now been corrected in the PDF and HTML versions of the Article.

