# Supplementary material for: Floats with bio-optical sensors reveal what processes trigger the North Atlantic bloom
Source: Nat Commun. 2018 Jan 15;9:190. doi: 10.1038/s41467-017-02143-6 (PMC5768750; doi:10.1038/s41467-017-02143-6)
Supplement: Supplementary file 1 — Supplementary Information [file 41467_2017_2143_MOESM1_ESM.pdf]

Supplementary Table 1. Relevant Information concerning the nine BGC-Argo floats used in this study.

wmo number	*Deployment location*	*Deployment Date*	*Sensor failure date*
6901480	-30.28°W, 62.76°N	16 June 2013	16 May 2017
6901482	-30.26°W, 62.76°N	17 June 2013	30 Jan 2015
6901485	-30.27°W, 62.76°N	16 June 2013	04 Mar 2016
6901486	-30.26°W, 62.76°N	16 June 2013	20 Jul 2017
6901489	-30.29°W, 62.76°N	16 June 2013	26 Feb 2015
6901516	-20.68°W, 62.77°N	17 Apr 2013	13 Sep 2016
6901524	-50.35°W, 58.68°N	15 May 2013	21 Jul 2017
6901525	-51.44°W, 57.78°N	14 May 2013	09 Oct 2016
6901527	-51.32°W, 57.79°N	14 May 2013	22 Apr 2017

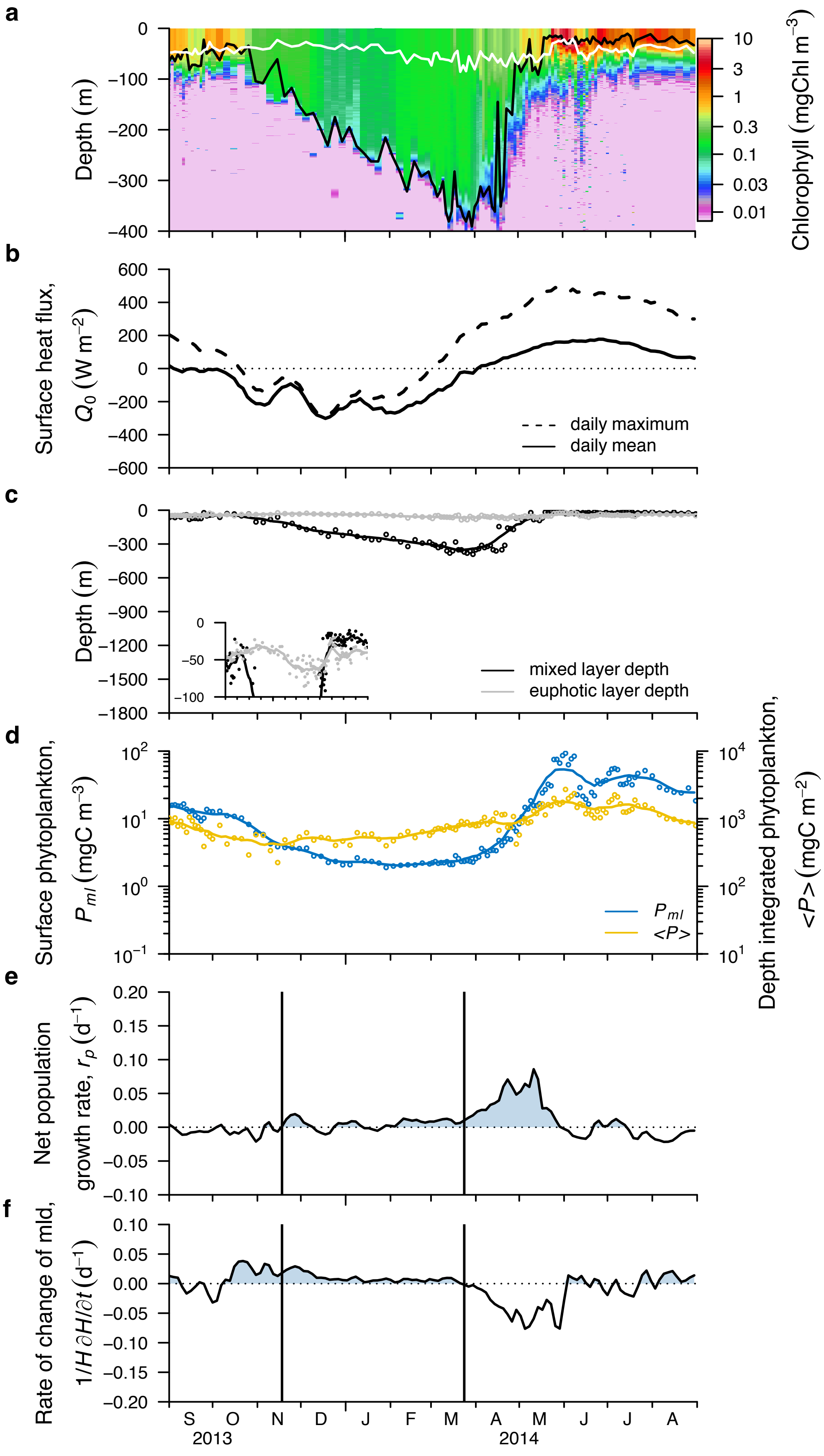
a
Depth (m)
Chlorophyll (mgChl m−3)
10
3
1
0.3
0.1
0.03
0.01
b
Surface heat flux,
Q0 (W m−2)
daily maximum
daily mean
c
Depth (m)
mixed layer depth
euphotic layer depth
d
Surface phytoplankton,
Pml (mgC m−3)
Depth integrated phytoplankton,
<P> (mgC m−2)
Pml
<P>
e
Net population
growth rate, rp (d−1)
f
Rate of change of mld,
1/H ∂H/∂t (d−1)
S
O
N
D
J
F
M
A
M
J
J
A
2013
2014

Supplementary Fig. 1. Time series of key variables measured or derived from float 6901516 from September 2013 to July 2014. (**a**) Vertical distribution of chlorophyll a concentration. The black and white continuous lines are the mixed layer, H and euphotic layer depths, H_e, respectively. Chlorophyll a values lower than the fluorometer detection level, ΔChl = 0.007 mg m^{-3}, were set to ΔChl. (**b**) Daily average surface heat flux (black continuous lines) and the maximum surface heat flux at 15h GMT, close to the local noon (black dashed lines). (**c**) Mixed layer (H, black circles) and euphotic layer depths (H_e, gray circles). A zoomed view of H and H_e for depths shallower than 100 m is included as an inset. (**d**) Mixed layer averaged phytoplankton carbon biomass concentration (P_{ml}, blue circles), and depth-integrated phytoplankton carbon biomass ($\langle P \rangle$, yellow circles). The lines in panels (**b**), (**c**) and (**d**) represent the running average over 24 days of the reported quantities. (**e**) Net population growth rate, r_p. (**f**) Rate of change of mixed layer depth, $\frac{1}{H}\frac{\partial H}{\partial t}$, computed from the 24-day average mixed layer depth. The first vertical line marks the initiation of the weak winter accumulation phase. It is computed as the time when r_p becomes positive for at least 24 days. The second vertical line marks the initiation of the spring bloom. It is computed as the time when $\frac{1}{H}\frac{\partial H}{\partial t}$ becomes negative for at least 24 days.

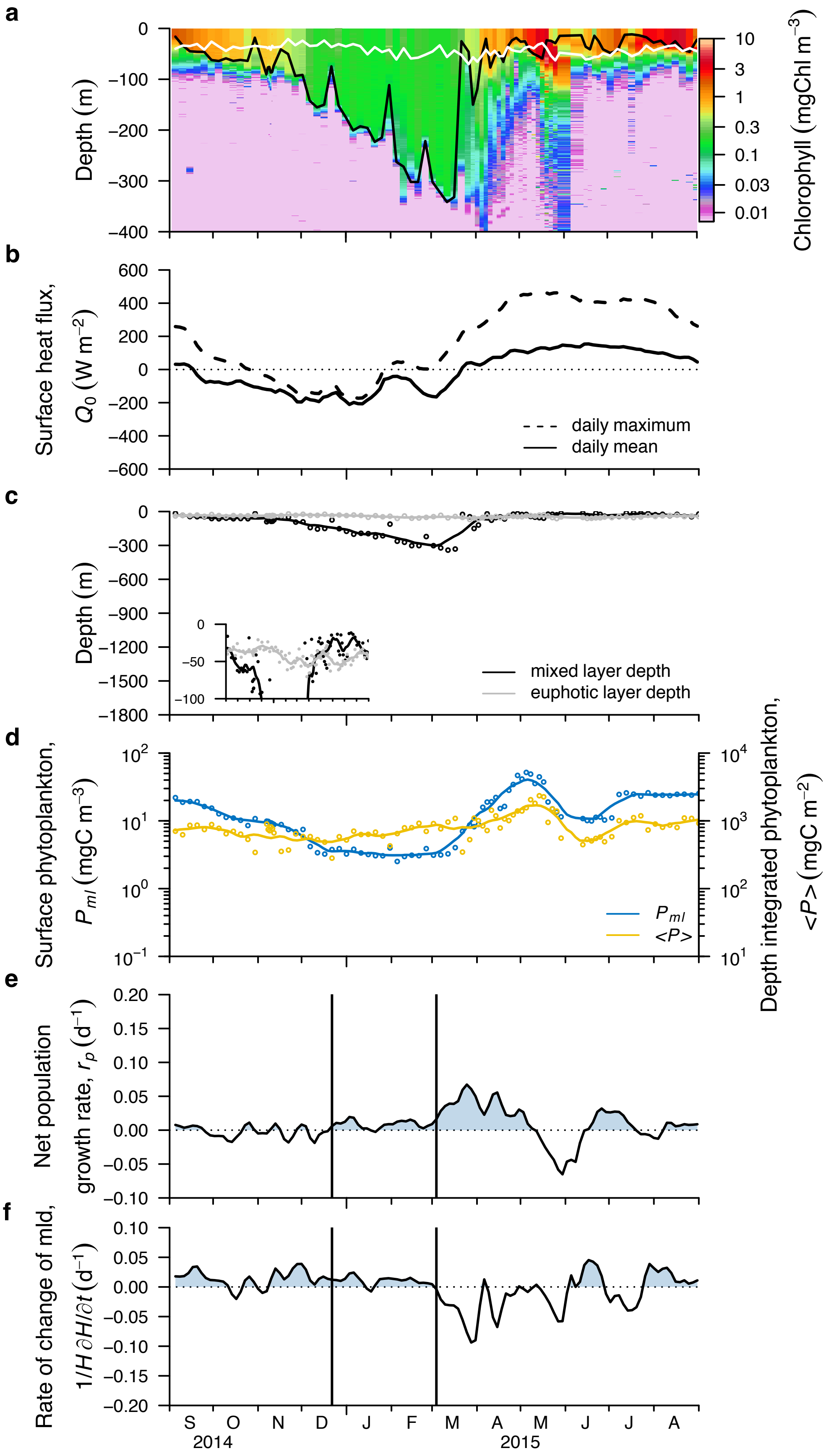
a
b
c
d
e
f
Depth (m)
Chlorophyll (mgChl m−3)
10
3
1
0.3
0.1
0.03
0.01
Surface heat flux, Q_0 (W m−2)
daily maximum
daily mean
mixed layer depth
euphotic layer depth
Surface phytoplankton, P_{ml} (mgC m−3)
Depth integrated phytoplankton, <P> (mgC m−2)
P_{ml}
<P>
Net population growth rate, r_p (d−1)
Rate of change of mld, $1/H\,\partial H/\partial t$ (d−1)
S O N D J F M A M J J A
2014
2015

1

Supplementary Fig. 2. Time series of key variables measured or derived from float 6901516 from September 2014 to July 2015. (**a**) Vertical distribution of chlorophyll a concentration. The black and white continuous lines are the mixed layer, H and euphotic layer depths, H_e, respectively. Chlorophyll a values lower than the fluorometer detection level, ΔChl = 0.007 mg m^{-3}, were set to ΔChl. (**b**) Daily average surface heat flux (black continuous lines) and the maximum surface heat flux at 15h GMT, close to the local noon (black dashed lines). (**c**) Mixed layer (H, black circles) and euphotic layer depths (H_e, gray circles). A zoomed view of H and H_e for depths shallower than 100 m is included as an inset. (**d**) Mixed layer averaged phytoplankton carbon biomass concentration (P_{ml}, blue circles), and depth-integrated phytoplankton carbon biomass ($\langle P \rangle$, yellow circles). The lines in panels (**b**), (**c**) and (**d**) represent the running average over 24 days of the reported quantities. (**e**) Net population growth rate, r_p. (**f**) Rate of change of mixed layer depth, $\frac{1}{H}\frac{\partial H}{\partial t}$, computed from the 24-day average mixed layer depth. The first vertical line marks the initiation of the weak winter accumulation phase. It is computed as the time when r_p becomes positive for at least 24 days. The second vertical line marks the initiation of the spring bloom. It is computed as the time when $\frac{1}{H}\frac{\partial H}{\partial t}$ becomes negative for at least 24 days.

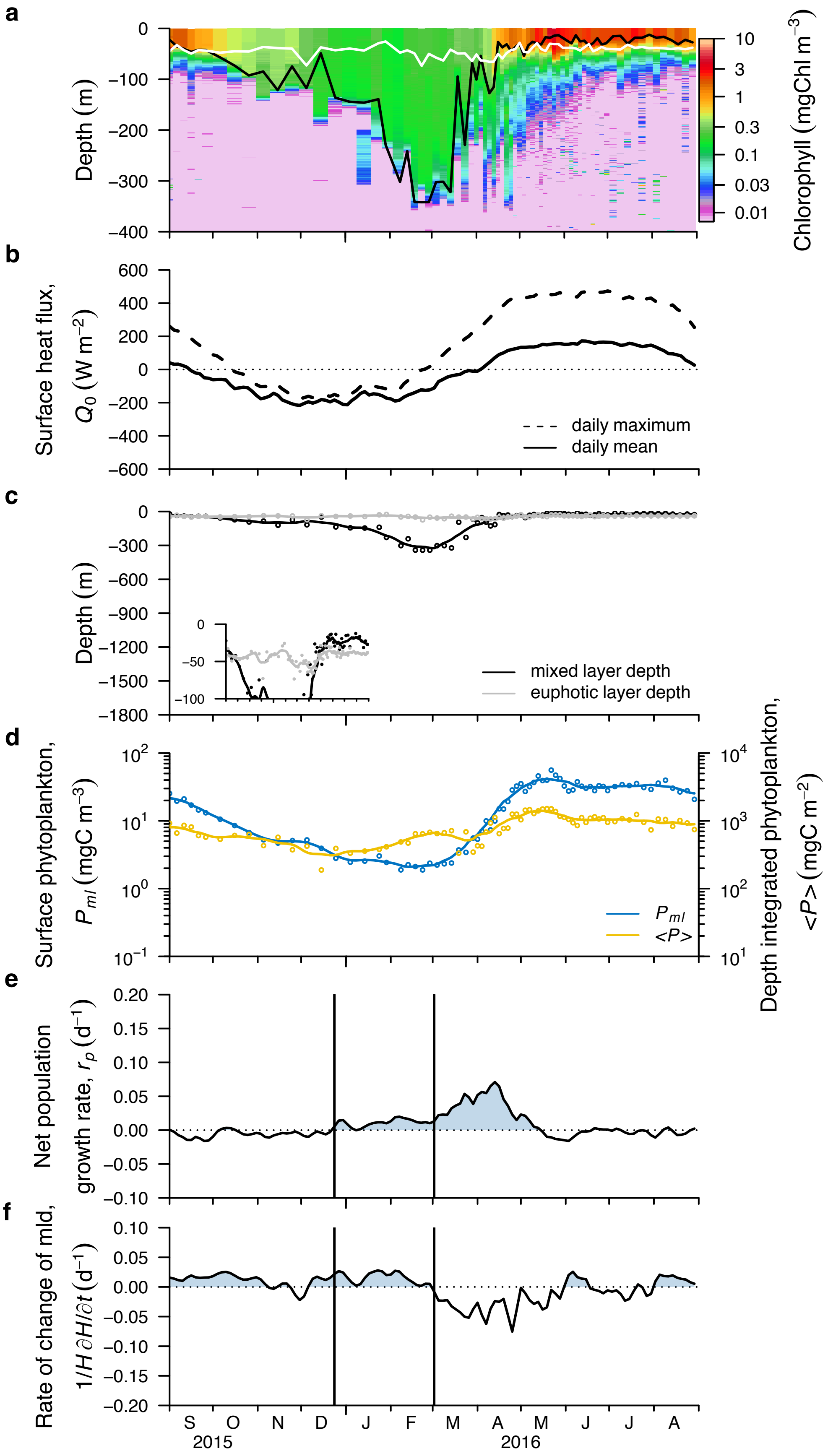
a
Depth (m)
Chlorophyll (mgChl m^{-3})
b
Surface heat flux, Q_0 (W m^{-2})
daily maximum
daily mean
c
Depth (m)
mixed layer depth
euphotic layer depth
d
Surface phytoplankton, P_{ml} (mgC m^{-3})
Depth integrated phytoplankton, <P> (mgC m^{-2})
P_{ml}
<P>
e
Net population growth rate, r_p (d^{-1})
f
Rate of change of mld, $1/H\,\partial H/\partial t$ (d^{-1})
S O N D J F M A M J J A
2015
2016

Supplementary Fig. 3. Time series of key variables measured or derived from float 6901516 from September 2015 to July 2016. (**a**) Vertical distribution of chlorophyll a concentration. The black and white continuous lines are the mixed layer, H and euphotic layer depths, H_e, respectively. Chlorophyll a values lower than the fluorometer detection level, ΔChl = 0.007 mg m^{-3}, were set to ΔChl. (**b**) Daily average surface heat flux (black continuous lines) and the maximum surface heat flux at 15h GMT, close to the local noon (black dashed lines). (**c**) Mixed layer (H, black circles) and euphotic layer depths (H_e, gray circles). A zoomed view of H and H_e for depths shallower than 100 m is included as an inset. (**d**) Mixed layer averaged phytoplankton carbon biomass concentration (P_{ml}, blue circles), and depth-integrated phytoplankton carbon biomass ($\langle P \rangle$, yellow circles). The lines in panels (**b**), (**c**) and (**d**) represent the running average over 24 days of the reported quantities. (**e**) Net population growth rate, r_p. (**f**) Rate of change of mixed layer depth, $\frac{1}{H}\frac{\partial H}{\partial t}$, computed from the 24-day average mixed layer depth. The first vertical line marks the initiation of the weak winter accumulation phase. It is computed as the time when r_p becomes positive for at least 24 days. The second vertical line marks the initiation of the spring bloom. It is computed as the time when $\frac{1}{H}\frac{\partial H}{\partial t}$ becomes negative for at least 24 days.

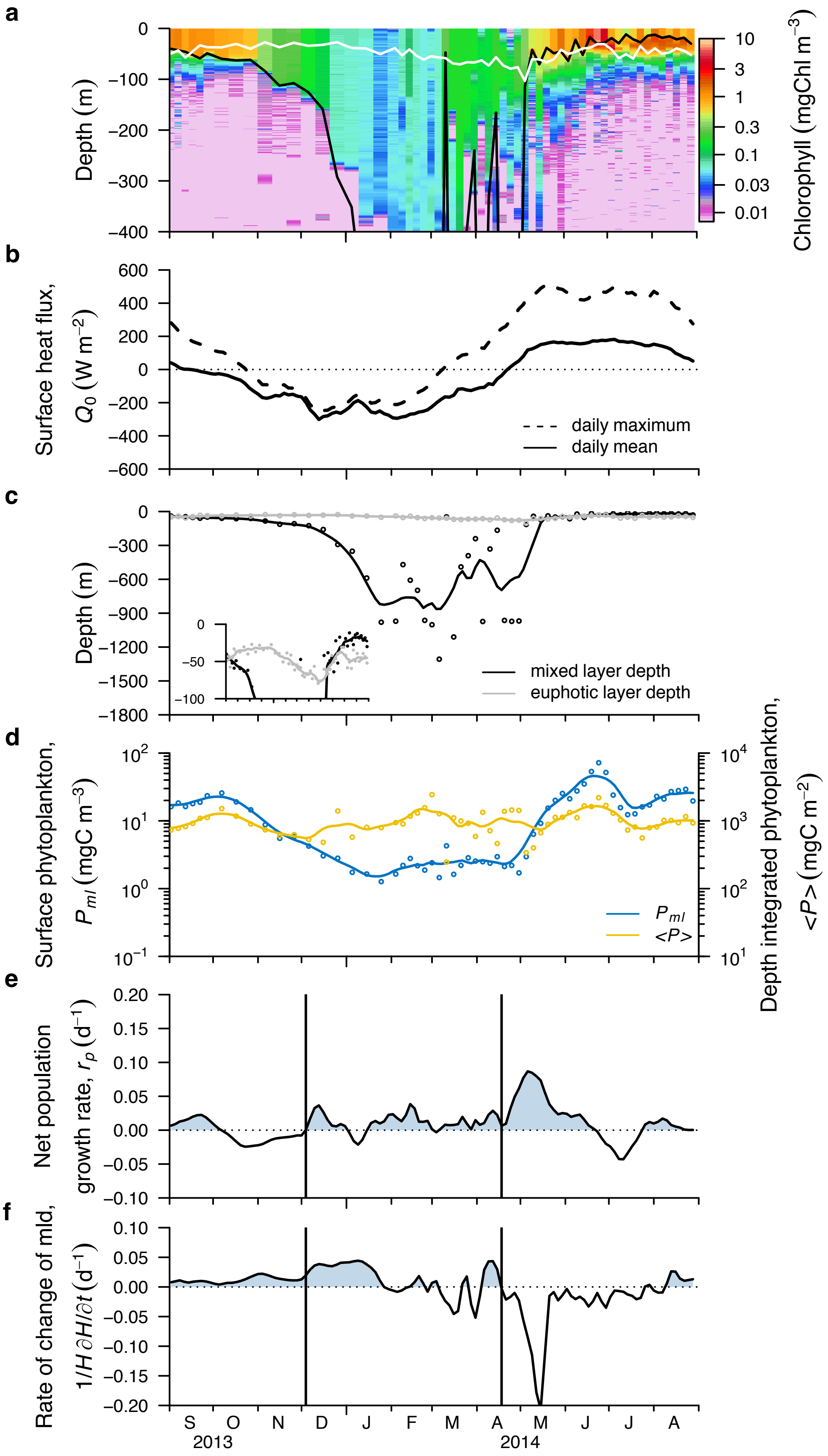
a
b
c
d
e
f
Depth (m)
Chlorophyll (mgChl m^{-3})
10
3
1
0.3
0.1
0.03
0.01
0
−100
−200
−300
−400
Surface heat flux,
Q_0 (W m^{-2})
600
400
200
−200
−400
−600
daily maximum
daily mean
−300
−600
−900
−1200
−1500
−1800
−50
−100
mixed layer depth
euphotic layer depth
Surface phytoplankton,
P_{ml} (mgC m^{-3})
10^2
10^1
10^0
10^{-1}
Depth integrated phytoplankton,
<P> (mgC m^{-2})
10^4
10^3
10^2
10^1
P_{ml}
<P>
Net population
growth rate, r_p (d^{-1})
0.20
0.15
0.10
0.05
0.00
−0.05
−0.10
Rate of change of mld,
$1/H\,\partial H/\partial t$ (d^{-1})
−0.15
−0.20
S
O
N
D
J
F
M
A
M
J
J
A
2013
2014

Supplementary Fig. 4. Time series of key variables measured or derived from float 6901524 from September 2013 to July 2014. (**a**) Vertical distribution of chlorophyll a concentration. The black and white continuous lines are the mixed layer, H and euphotic layer depths, H_e, respectively. Chlorophyll a values lower than the fluorometer detection level, ΔChl = 0.007 mg m^{-3}, were set to ΔChl. (**b**) Daily average surface heat flux (black continuous lines) and the maximum surface heat flux at 15h GMT, close to the local noon (black dashed lines). (**c**) Mixed layer (H, black circles) and euphotic layer depths (H_e, gray circles). A zoomed view of H and H_e for depths shallower than 100 m is included as an inset. (**d**) Mixed layer averaged phytoplankton carbon biomass concentration (P_{ml}, blue circles), and depth-integrated phytoplankton carbon biomass ($\langle P \rangle$, yellow circles). The lines in panels (**b**), (**c**) and (**d**) represent the running average over 24 days of the reported quantities. (**e**) Net population growth rate, r_p. (**f**) Rate of change of mixed layer depth, $\frac{1}{H}\frac{\partial H}{\partial t}$, computed from the 24-day average mixed layer depth. The first vertical line marks the initiation of the weak winter accumulation phase. It is computed as the time when r_p becomes positive for at least 24 days. The second vertical line marks the initiation of the spring bloom. It is computed as the time when $\frac{1}{H}\frac{\partial H}{\partial t}$ becomes negative for at least 24 days.

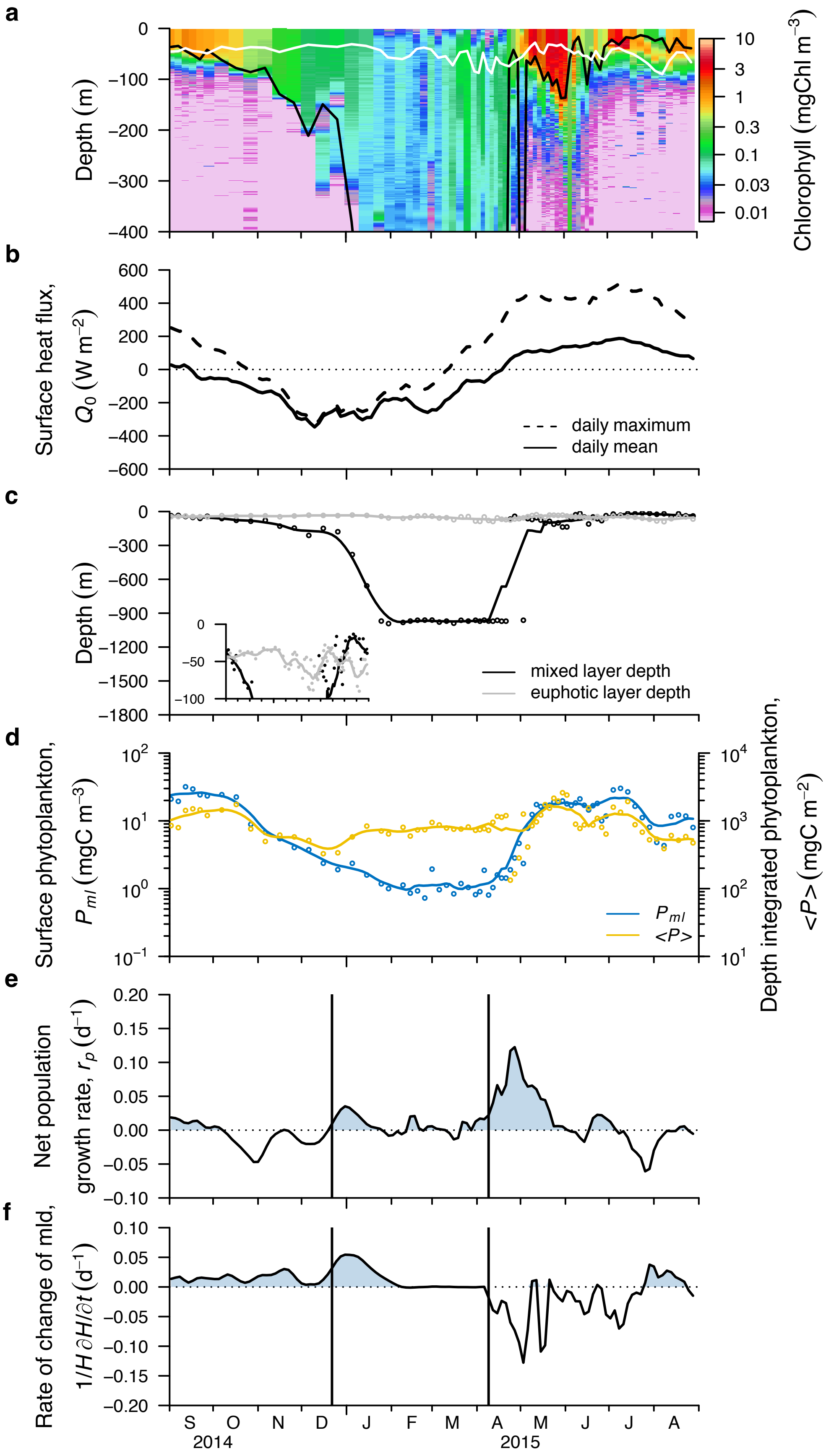
a
Depth (m)
0
−100
−200
−300
−400
Chlorophyll (mgChl m^{-3})
10
3
1
0.3
0.1
0.03
0.01
b
Surface heat flux,
Q_0 (W m^{-2})
600
400
200
0
−200
−400
−600
daily maximum
daily mean
c
Depth (m)
0
−300
−600
−900
−1200
−1500
−1800
0
−50
−100
mixed layer depth
euphotic layer depth
d
Surface phytoplankton,
P_{ml} (mgC m^{-3})
10^2
10^1
10^0
10^{-1}
Depth integrated phytoplankton,
<P> (mgC m^{-2})
10^4
10^3
10^2
10^1
P_{ml}
<P>
e
Net population
growth rate, r_p (d^{-1})
0.20
0.15
0.10
0.05
0.00
−0.05
−0.10
f
Rate of change of mld,
$1/H\,\partial H/\partial t$ (d^{-1})
0.10
0.05
0.00
−0.05
−0.10
−0.15
−0.20
S
O
N
D
J
F
M
A
M
J
J
A
2014
2015

Supplementary Fig. 5. Time series of key variables measured or derived from float 6901524 from September 2014 to July 2015. (**a**) Vertical distribution of chlorophyll a concentration. The black and white continuous lines are the mixed layer, H and euphotic layer depths, H_e, respectively. Chlorophyll a values lower than the fluorometer detection level, ΔChl = 0.007 mg m^{-3}, were set to ΔChl. (**b**) Daily average surface heat flux (black continuous lines) and the maximum surface heat flux at 15h GMT, close to the local noon (black dashed lines). (**c**) Mixed layer (H, black circles) and euphotic layer depths (H_e, gray circles). A zoomed view of H and H_e for depths shallower than 100 m is included as an inset. (**d**) Mixed layer averaged phytoplankton carbon biomass concentration (P_{ml}, blue circles), and depth-integrated phytoplankton carbon biomass ($\langle P \rangle$, yellow circles). The lines in panels (**b**), (**c**) and (**d**) represent the running average over 24 days of the reported quantities. (**e**) Net population growth rate, r_p. (**f**) Rate of change of mixed layer depth, $\frac{1}{H}\frac{\partial H}{\partial t}$, computed from the 24-day average mixed layer depth. The first vertical line marks the initiation of the weak winter accumulation phase. It is computed as the time when r_p becomes positive for at least 24 days. The second vertical line marks the initiation of the spring bloom. It is computed as the time when $\frac{1}{H}\frac{\partial H}{\partial t}$ becomes negative for at least 24 days. Note that from February to April, the float did not profile deep enough to sample the base of the mixed layer. As a result, there is certainly a bias in the estimates of

μ_p, r_p, and $\frac{1}{H}\frac{\partial H}{\partial t}$ .These values were therefore excluded from the calculations of the median μ_p, r_p, and $\frac{1}{H}\frac{\partial H}{\partial t}$ (Fig. 3 in the main paper).

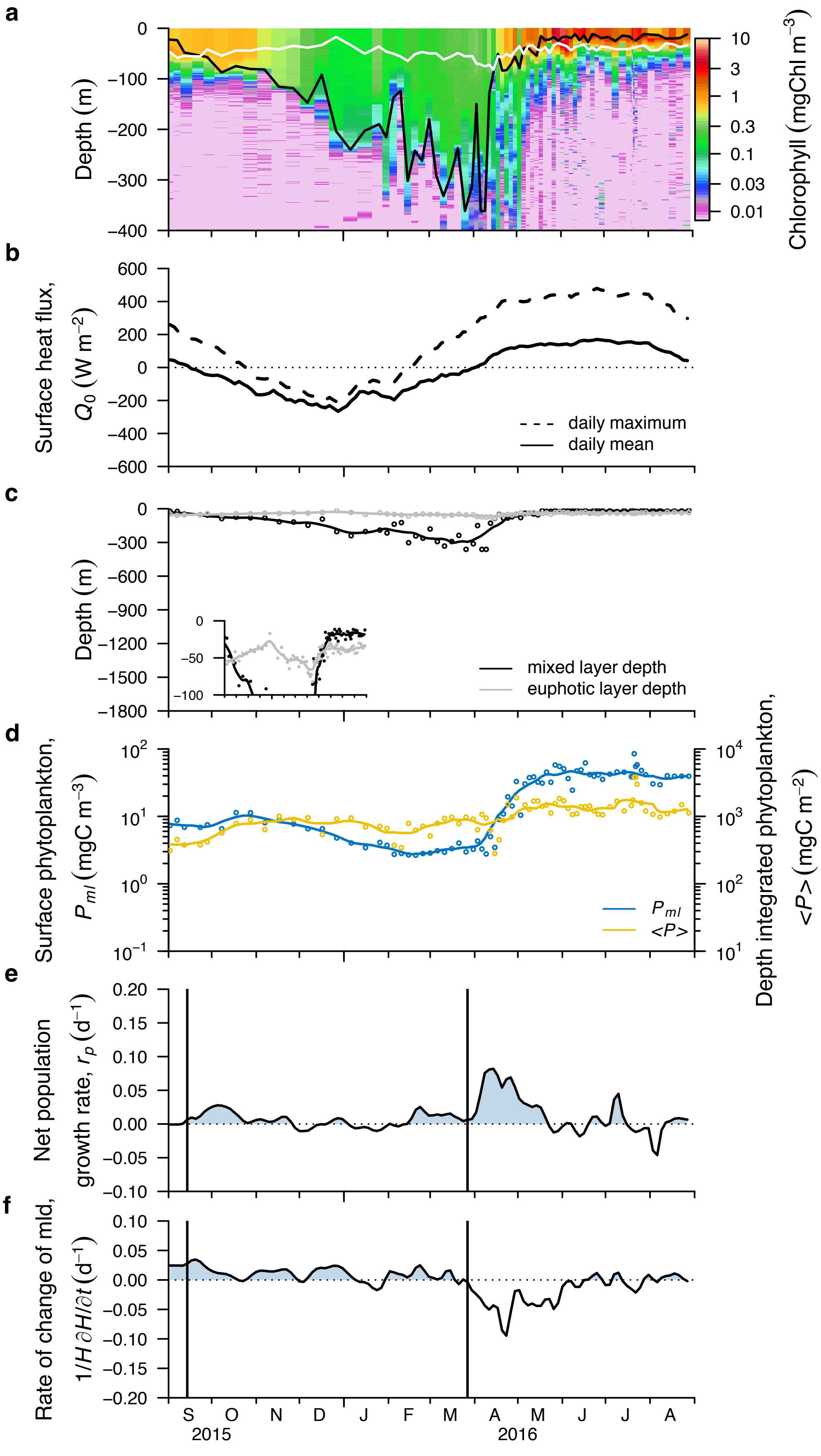
a
Depth (m)
Chlorophyll (mgChl m^{-3})
10
3
1
0.3
0.1
0.03
0.01
b
Surface heat flux,
Q_0 (W m^{-2})
daily maximum
daily mean
c
Depth (m)
mixed layer depth
euphotic layer depth
d
Surface phytoplankton,
P_{ml} (mgC m^{-3})
Depth integrated phytoplankton,
<P> (mgC m^{-2})
P_{ml}
<P>
e
Net population
growth rate, r_p (d^{-1})
f
Rate of change of mld,
$1/H\,\partial H/\partial t$ (d^{-1})
S
O
N
D
J
F
M
A
M
J
J
A
2015
2016

Supplementary Fig. 6. Time series of key variables measured or derived from float 6901524 from September 2015 to July 2016. (**a**) Vertical distribution of chlorophyll a concentration. The black and white continuous lines are the mixed layer, H and euphotic layer depths, H_e, respectively. Chlorophyll a values lower than the fluorometer detection level, ΔChl = 0.007 mg m^{-3}, were set to ΔChl. (**b**) Daily average surface heat flux (black continuous lines) and the maximum surface heat flux at 15h GMT, close to the local noon (black dashed lines). (**c**) Mixed layer (H, black circles) and euphotic layer depths (H_e, gray circles). A zoomed view of H and H_e for depths shallower than 100 m is included as an inset. (**d**) Mixed layer averaged phytoplankton carbon biomass concentration (P_{ml}, blue circles), and depth-integrated phytoplankton carbon biomass ($\langle P \rangle$, yellow circles). The lines in panels (**b**), (**c**) and (**d**) represent the running average over 24 days of the reported quantities. (**e**) Net population growth rate, r_p. (**f**) Rate of change of mixed layer depth, $\frac{1}{H}\frac{\partial H}{\partial t}$, computed from the 24-day average mixed layer depth. The first vertical line marks the initiation of the weak winter accumulation phase. It is computed as the time when r_p becomes positive for at least 24 days. The second vertical line marks the initiation of the spring bloom. It is computed as the time when $\frac{1}{H}\frac{\partial H}{\partial t}$ becomes negative for at least 24 days.

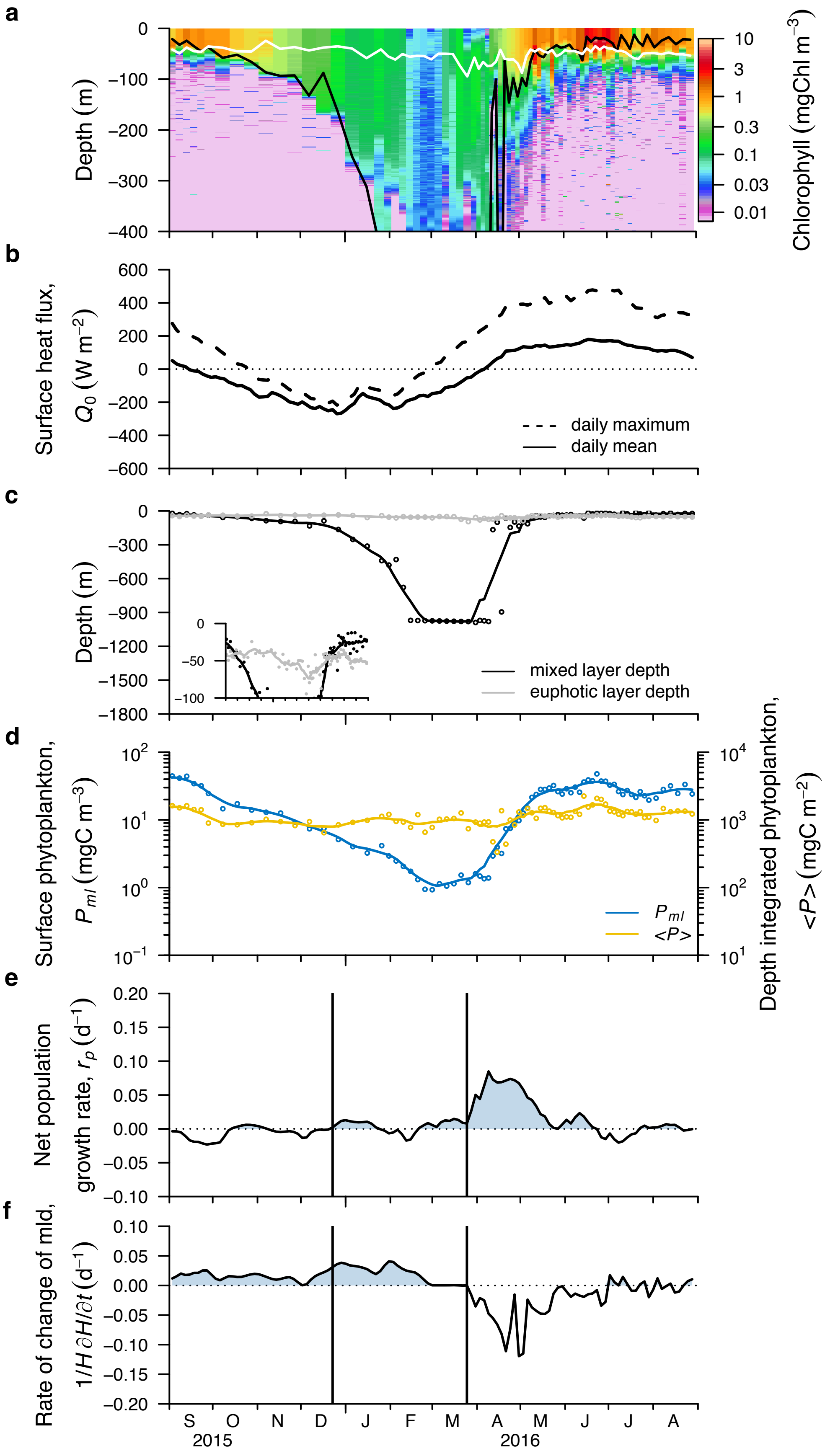
a
Depth (m)
0
−100
−200
−300
−400
Chlorophyll (mgChl m−3)
10
3
1
0.3
0.1
0.03
0.01
b
Surface heat flux,
Q0 (W m−2)
600
400
200
0
−200
−400
−600
daily maximum
daily mean
c
Depth (m)
0
−300
−600
−900
−1200
−1500
−1800
0
−50
−100
mixed layer depth
euphotic layer depth
d
Surface phytoplankton,
Pml (mgC m−3)
10^2
10^1
10^0
10^−1
Depth integrated phytoplankton,
<P> (mgC m−2)
10^4
10^3
10^2
10^1
Pml
<P>
e
Net population
growth rate, rp (d−1)
0.20
0.15
0.10
0.05
0.00
−0.05
−0.10
f
Rate of change of mld,
1/H ∂H/∂t (d−1)
0.10
0.05
0.00
−0.05
−0.10
−0.15
−0.20
S
O
N
D
J
F
M
A
M
J
J
A
2015
2016

Supplementary Fig. 7. Time series of key variables measured or derived from float 6901527 from September 2015 to July 2016. (**a**) Vertical distribution of chlorophyll a concentration. The black and white continuous lines are the mixed layer, H and euphotic layer depths, H_e, respectively. Chlorophyll a values lower than the fluorometer detection level, ΔChl = 0.007 mg m^{-3}, were set to ΔChl. (**b**) Daily average surface heat flux (black continuous lines) and the maximum surface heat flux at 15h GMT, close to the local noon (black dashed lines). (**c**) Mixed layer (H, black circles) and euphotic layer depths (H_e, gray circles). A zoomed view of H and H_e for depths shallower than 100 m is included as an inset. (**d**) Mixed layer averaged phytoplankton carbon biomass concentration (P_{ml}, blue circles), and depth-integrated phytoplankton carbon biomass ($\langle P \rangle$, yellow circles). The lines in panels (**b**), (**c**) and (**d**) represent the running average over 24 days of the reported quantities. (**e**) Net population growth rate, r_p. (**f**) Rate of change of mixed layer depth, $\frac{1}{H}\frac{\partial H}{\partial t}$, computed from the 24-day average mixed layer depth. The first vertical line marks the initiation of the weak winter accumulation phase. It is computed as the time when r_p becomes positive for at least 24 days. The second vertical line marks the initiation of the spring bloom. It is computed as the time when $\frac{1}{H}\frac{\partial H}{\partial t}$ becomes negative for at least 24 days. Note that from February to March, the float did not profile deep enough to sample the base of the mixed layer. As a result, there is certainly a bias in the estimates

of μ_p, r_p, and $\frac{1}{H}\frac{\partial H}{\partial t}$. These values were therefore excluded from the calculations of the median μ_p, r_p, and $\frac{1}{H}\frac{\partial H}{\partial t}$ (Fig. 3 in the main paper).

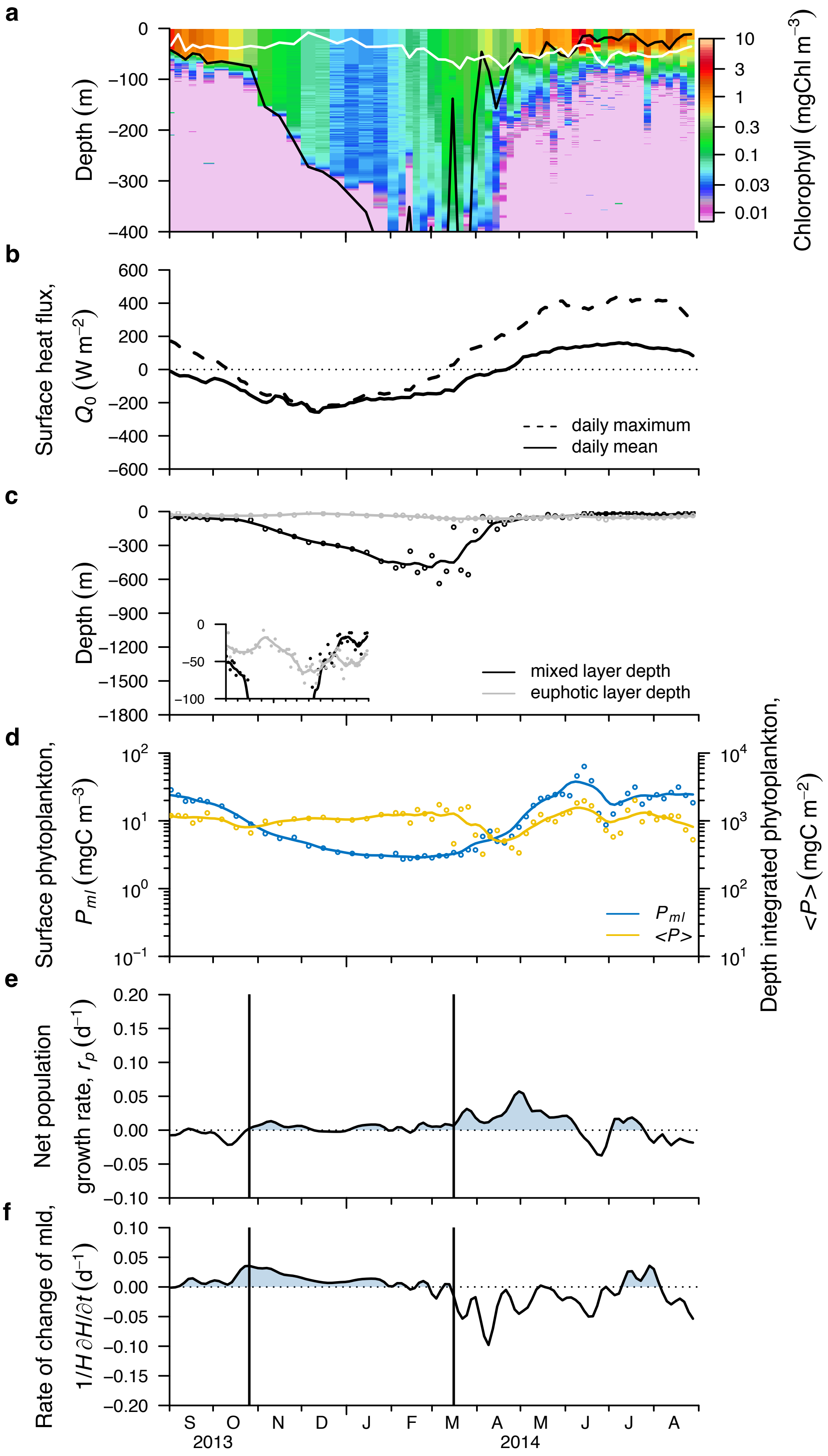

a
0
−100
−200
−300
−400
Depth (m)
10
3
1
0.3
0.1
0.03
0.01
Chlorophyll (mgChl m−3)
b
Surface heat flux, Q0 (W m−2)
600
400
200
0
−200
−400
−600
daily maximum
daily mean
c
Depth (m)
0
−300
−600
−900
−1200
−1500
−1800
0
−50
−100
mixed layer depth
euphotic layer depth
d
Surface phytoplankton, Pml (mgC m−3)
Depth integrated phytoplankton, <P> (mgC m−2)
Pml
<P>
e
Net population growth rate, rp (d−1)
0.20
0.15
0.10
0.05
0.00
−0.05
−0.10
f
Rate of change of mld, 1/H ∂H/∂t (d−1)
0.10
0.05
0.00
−0.05
−0.10
−0.15
−0.20
S
O
N
D
J
F
M
A
M
J
J
A
2013
2014

Supplementary Fig. 8. Time series of key variables measured or derived from float 6901485 from September 2013 to July 2014. (**a**) Vertical distribution of chlorophyll a concentration. The black and white continuous lines are the mixed layer, H and euphotic layer depths, H_e, respectively. Chlorophyll a values lower than the fluorometer detection level, ΔChl = 0.007 mg m^{-3}, were set to ΔChl. (**b**) Daily average surface heat flux (black continuous lines) and the maximum surface heat flux at 15h GMT, close to the local noon (black dashed lines). (**c**) Mixed layer (H, black circles) and euphotic layer depths (H_e, gray circles). A zoomed view of H and H_e for depths shallower than 100 m is included as an inset. (**d**) Mixed layer averaged phytoplankton carbon biomass concentration (P_{ml}, blue circles), and depth-integrated phytoplankton carbon biomass ($\langle P \rangle$, yellow circles). The lines in panels (**b**), (**c**) and (**d**) represent the running average over 24 days of the reported quantities. (**e**) Net population growth rate, r_p. (**f**) Rate of change of mixed layer depth, $\frac{1}{H}\frac{\partial H}{\partial t}$, computed from the 24-day average mixed layer depth. The first vertical line marks the initiation of the weak winter accumulation phase. It is computed as the time when r_p becomes positive for at least 24 days. The second vertical line marks the

initiation of the spring bloom. It is computed as the time when $\frac{1}{H}\frac{\partial H}{\partial t}$ becomes negative for at least 24 days.

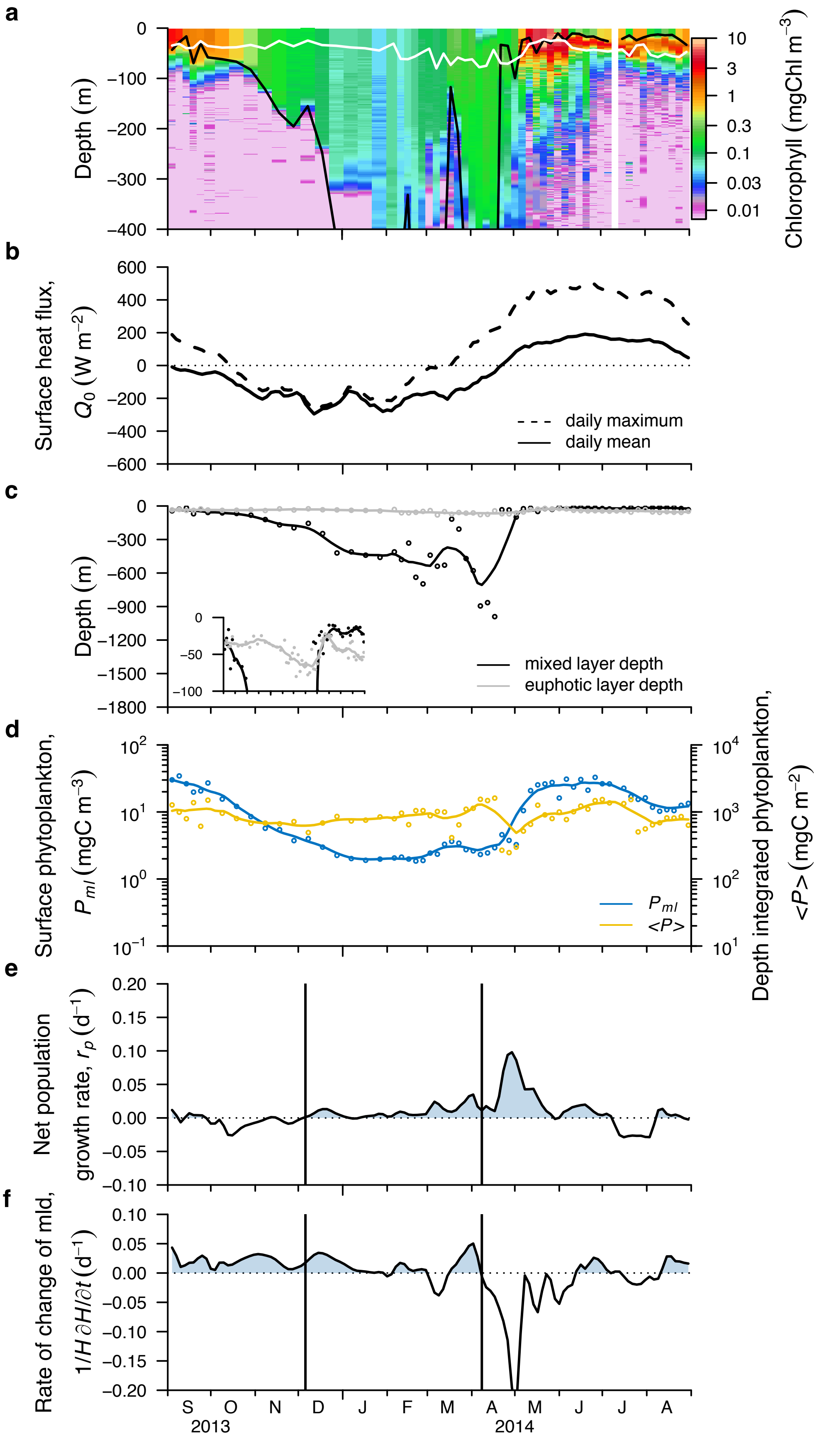

a
b
c
d
e
f
Depth (m)
Chlorophyll (mgChl m−3)
10
3
1
0.3
0.1
0.03
0.01
Surface heat flux,
Q0 (W m−2)
daily maximum
daily mean
mixed layer depth
euphotic layer depth
Surface phytoplankton,
Pml (mgC m−3)
Depth integrated phytoplankton,
<P> (mgC m−2)
Pml
<P>
Net population
growth rate, rp (d−1)
Rate of change of mld,
1/H ∂H/∂t (d−1)
S O N D J F M A M J J A
2013
2014

Supplementary Fig. 9. Time series of key variables measured or derived from float 6901486 from September 2013 to July 2014. (**a**) Vertical distribution of chlorophyll a concentration. The black and white continuous lines are the mixed layer, H and euphotic layer depths, H_e, respectively. Chlorophyll a values lower than the fluorometer detection level, ΔChl = 0.007 mg m^{-3}, were set to ΔChl. (**b**) Daily average surface heat flux (black continuous lines) and the maximum surface heat flux at 15h GMT, close to the local noon (black dashed lines). (**c**) Mixed layer (H, black circles) and euphotic layer depths (H_e, gray circles). A zoomed view of H and H_e for depths shallower than 100 m is included as an inset. (**d**) Mixed layer averaged phytoplankton carbon biomass concentration (P_{ml}, blue circles), and depth-integrated phytoplankton carbon biomass ($\langle P \rangle$, yellow circles). The lines in panels (**b**), (**c**) and (**d**) represent the running average over 24 days of the reported quantities. (**e**) Net population growth rate, r_p. (**f**) Rate of change of mixed layer depth, $\frac{1}{H}\frac{\partial H}{\partial t}$, computed from the 24-day average mixed layer depth. The first vertical line marks the initiation of the weak winter accumulation phase. It is computed as the time when r_p becomes positive for at least 24 days. The second vertical line marks the initiation of the spring bloom. It is computed as the time when $\frac{1}{H}\frac{\partial H}{\partial t}$ becomes negative for at least 24 days. A freshening of the surface waters was observed several weeks after the onset of stratification. These changes in surface waters properties did not impact our study, because the freshening occurred well after the onset of the spring bloom.

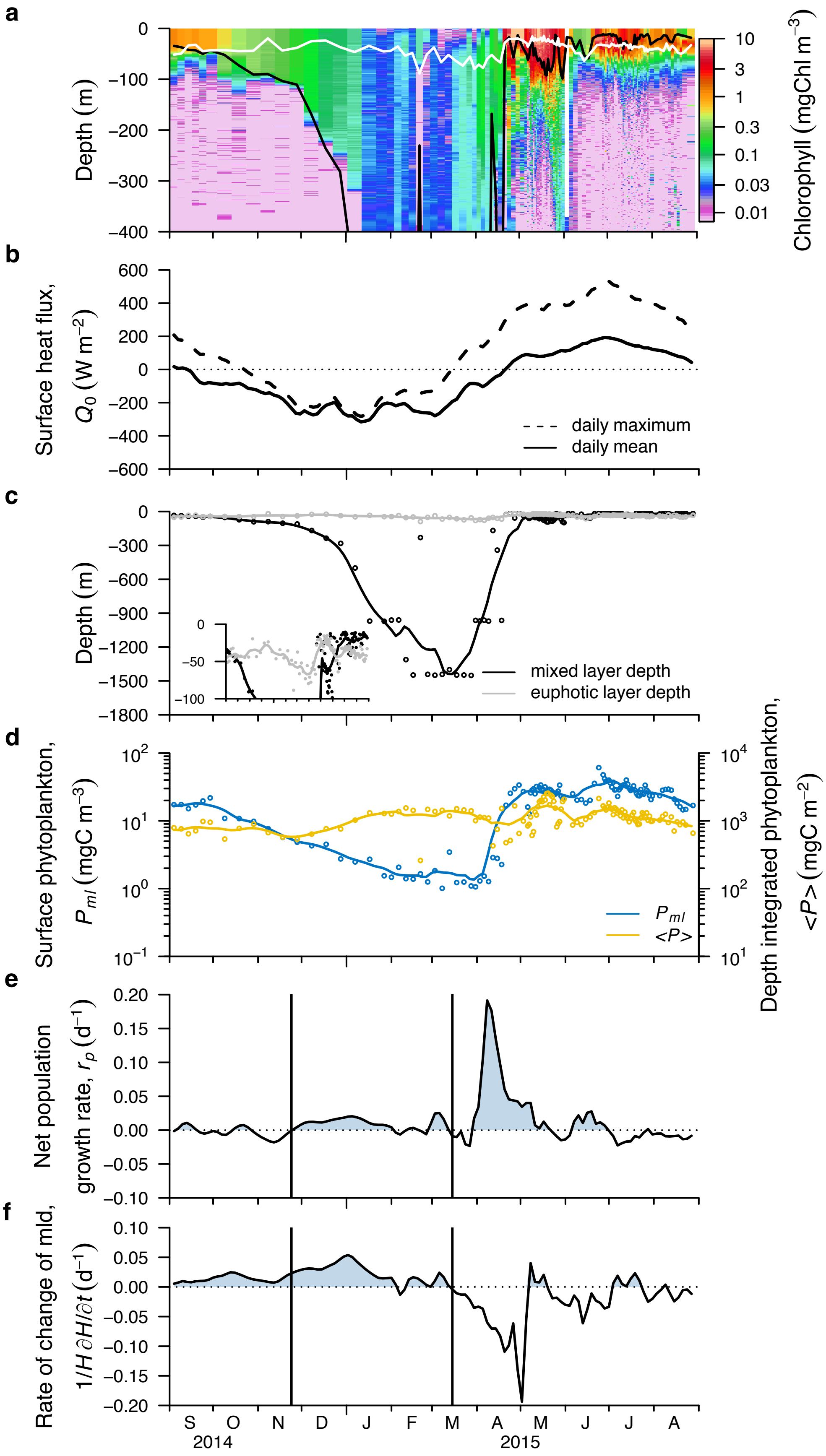
a
0
−100
−200
−300
−400
Depth (m)
10
3
1
0.3
0.1
0.03
0.01
Chlorophyll (mgChl m−3)
b
600
400
200
0
−200
−400
−600
Surface heat flux,
Q0 (W m−2)
daily maximum
daily mean
c
0
−300
−600
−900
−1200
−1500
−1800
Depth (m)
0
−50
−100
mixed layer depth
euphotic layer depth
d
10^2
10^1
10^0
10^−1
Surface phytoplankton,
Pml (mgC m−3)
10^4
10^3
10^2
10^1
Depth integrated phytoplankton,
<P> (mgC m−2)
Pml
<P>
e
0.20
0.15
0.10
0.05
0.00
−0.05
−0.10
Net population
growth rate, rp (d−1)
f
0.10
0.05
0.00
−0.05
−0.10
−0.15
−0.20
Rate of change of mld,
1/H ∂H/∂t (d−1)
S
O
N
D
J
F
M
A
M
J
J
A
2014
2015

1

Supplementary Fig. 10. Time series of key variables measured or derived from float 6901486 from September 2014 to July 2015. (**a**) Vertical distribution of chlorophyll a concentration. The black and white continuous lines are the mixed layer, H and euphotic layer depths, H_e, respectively. Chlorophyll a values lower than the fluorometer detection level, ΔChl = 0.007 mg m^{-3}, were set to ΔChl. (**b**) Daily average surface heat flux (black continuous lines) and the maximum surface heat flux at 15h GMT, close to the local noon (black dashed lines). (**c**) Mixed layer (H, black circles) and euphotic layer depths (H_e, gray circles). A zoomed view of H and H_e for depths shallower than 100 m is included as an inset. (**d**) Mixed layer averaged phytoplankton carbon biomass concentration (P_{ml}, blue circles), and depth-integrated phytoplankton carbon biomass ($\langle P \rangle$, yellow circles). The lines in panels (**b**), (**c**) and (**d**) represent the running average over 24 days of the reported quantities. (**e**) Net population growth rate, r_p. (**f**) Rate of change of mixed layer depth, $\frac{1}{H}\frac{\partial H}{\partial t}$, computed from the 24-day average mixed layer depth. The first vertical line marks the initiation of the weak winter accumulation phase. It is computed as the time when r_p becomes positive for at least 24 days. The second vertical line marks the initiation of the spring bloom. It is computed as the time when $\frac{1}{H}\frac{\partial H}{\partial t}$ becomes negative for at least 24 days. Note that from February to March, the float did not profile deep enough to sample the base of the mixed layer. As a result, there is certainly a bias in the estimates of μ_p, r_p, and $\frac{1}{H}\frac{\partial H}{\partial t}$. These values were therefore excluded from the calculations of the median μ_p, r_p, and $\frac{1}{H}\frac{\partial H}{\partial t}$ (Fig. 3 in the main paper). Moreover, a freshening of the surface waters was observed several weeks after the onset of spring restratification. These

changes in surface waters properties did not impact our study, because the freshening occurred well after the onset of the spring bloom.

1

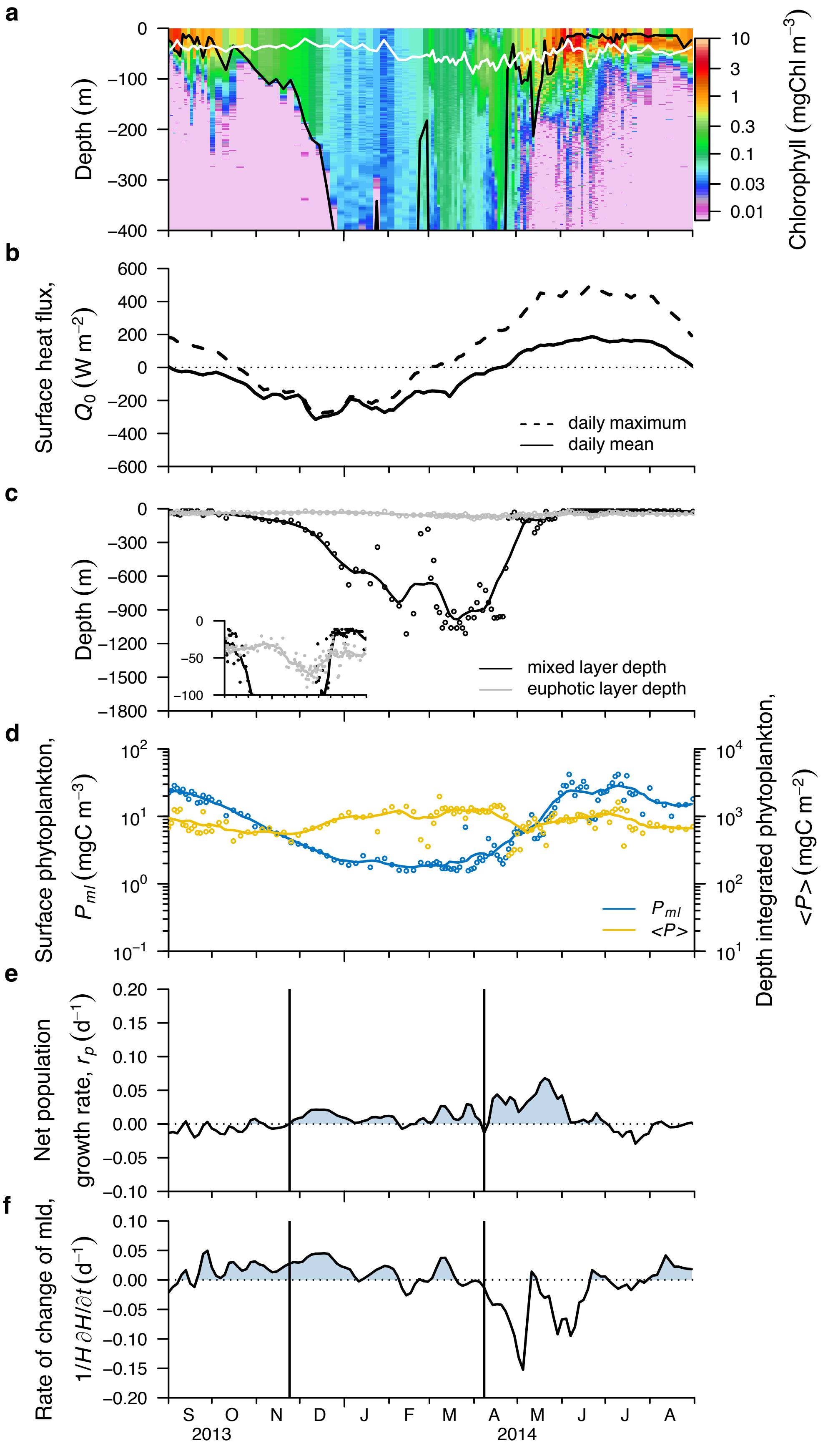

a
b
c
d
e
f
Depth (m)
Chlorophyll (mgChl m^{-3})
Surface heat flux, Q_0 (W m^{-2})
daily maximum
daily mean
Depth (m)
mixed layer depth
euphotic layer depth
Surface phytoplankton, P_{ml} (mgC m^{-3})
Depth integrated phytoplankton, <P> (mgC m^{-2})
P_{ml}
<P>
Net population growth rate, r_p (d^{-1})
Rate of change of mld, $1/H\,\partial H/\partial t$ (d^{-1})
S O N D J F M A M J J A
2013
2014

Supplementary Fig. 11. Time series of key variables measured or derived from float 6901480 from September 2013 to July 2014. (**a**) Vertical distribution of chlorophyll a concentration. The black and white continuous lines are the mixed layer, H and euphotic layer depths, H_e, respectively. Chlorophyll a values lower than the fluorometer detection level, ΔChl = 0.007 mg m^{-3}, were set to ΔChl. (**b**) Daily average surface heat flux (black continuous lines) and the maximum surface heat flux at 15h GMT, close to the local noon (black dashed lines). (**c**) Mixed layer (H, black circles) and euphotic layer depths (H_e, gray circles). A zoomed view of H and H_e for depths shallower than 100 m is included as an inset. (**d**) Mixed layer averaged phytoplankton carbon biomass concentration (P_{ml}, blue circles), and depth-integrated phytoplankton carbon biomass ($\langle P \rangle$, yellow circles). The lines in panels (**b**), (**c**) and (**d**) represent the running average over 24 days of the reported quantities. (**e**) Net population growth rate, r_p. (**f**) Rate of change of mixed layer depth, $\frac{1}{H}\frac{\partial H}{\partial t}$, computed from the 24-day average mixed layer depth. The first vertical line marks the initiation of the weak winter accumulation phase. It is computed as the time when r_p becomes positive for at least 24 days. The second vertical line marks the initiation of the spring bloom. It is computed as the time when $\frac{1}{H}\frac{\partial H}{\partial t}$ becomes negative for at least 24 days. A freshening of the surface waters was observed several weeks after the onset of spring restratification. These changes in surface waters properties did not impact our study, because the freshening occurred well after the onset of the spring bloom.

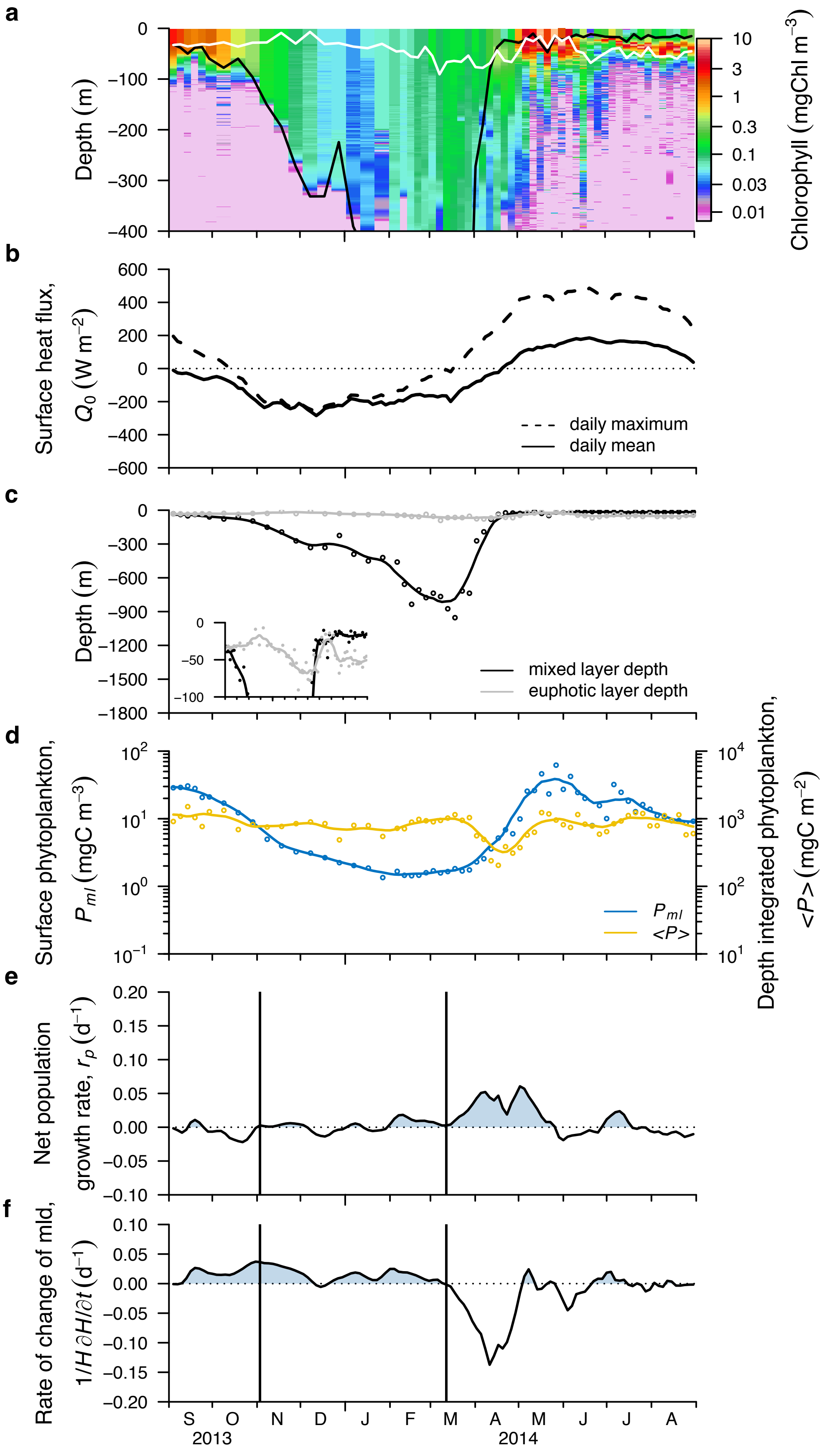
a
b
c
d
e
f
Depth (m)
Chlorophyll (mgChl m^{-3})
10
3
1
0.3
0.1
0.03
0.01
Surface heat flux, Q_0 (W m^{-2})
daily maximum
daily mean
Depth (m)
mixed layer depth
euphotic layer depth
Surface phytoplankton, P_{ml} (mgC m^{-3})
Depth integrated phytoplankton, <P> (mgC m^{-2})
P_{ml}
<P>
Net population growth rate, r_p (d^{-1})
Rate of change of mld, $1/H\,\partial H/\partial t$ (d^{-1})
S
O
N
D
J
F
M
A
M
J
J
A
2013
2014

Supplementary Fig. 12. Time series of key variables measured or derived from float 6901489 from September 2013 to July 2014. (**a**) Vertical distribution of chlorophyll a concentration. The black and white continuous lines are the mixed layer, H and euphotic layer depths, H_e, respectively. Chlorophyll a values lower than the fluorometer detection level, ΔChl = 0.007 mg m^{-3}, were set to ΔChl. (**b**) Daily average surface heat flux (black continuous lines) and the maximum surface heat flux at 15h GMT, close to the local noon (black dashed lines). (**c**) Mixed layer (H, black circles) and euphotic layer depths (H_e, gray circles). A zoomed view of H and H_e for depths shallower than 100 m is included as an inset. (**d**) Mixed layer averaged phytoplankton carbon biomass concentration (P_{ml}, blue circles), and depth-integrated phytoplankton carbon biomass ($\langle P \rangle$, yellow circles). The lines in panels (**b**), (**c**) and (**d**) represent the running average over 24 days of the reported quantities. (**e**) Net population growth rate, r_p. (**f**) Rate of change of mixed layer depth, $\frac{1}{H}\frac{\partial H}{\partial t}$, computed from the 24-day average mixed layer depth. The first vertical line marks the initiation of the weak winter accumulation phase. It is computed as the time when r_p becomes positive for at least 24 days. The second vertical line marks the initiation of the spring bloom. It is computed as the time when $\frac{1}{H}\frac{\partial H}{\partial t}$ becomes negative for at least 24 days. A freshening of the surface waters was observed several weeks after the onset of spring restratification. These changes in surface waters properties did not impact our study, because the freshening occurred well after the onset of the spring bloom.

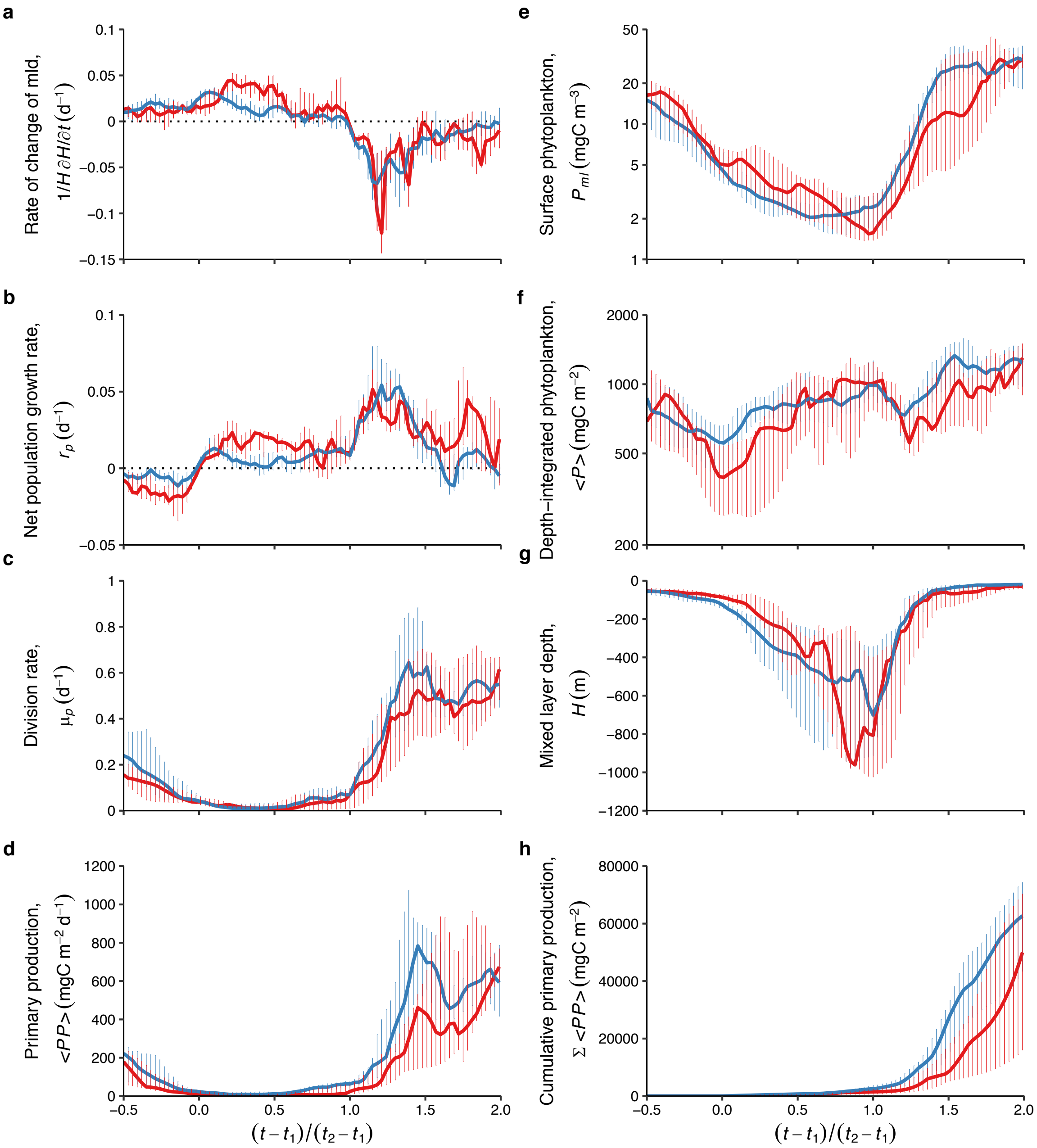

Supplementary Fig. 13. Comparison between the 12 quasi one-dimensional time series with the seven time series where a transient restratification was observed while the air-sea heat fluxes were still negative. The blue lines represent the 12 time series where a one-dimensional description of the bloom dynamics can be assumed. The red lines represent the seven time series where a transient restratification was observed

while the air-sea heat fluxes were still negative. Median (solid thick line) and interquartile range (vertical bars) of the rate of change of mixed layer depth $\frac{1}{H}\frac{\partial H}{\partial t}$ (panel **a**), the net population growth rate r_p (panel **b**), phytoplankton division rates μ_p (panel **c**), the daily-averaged and depth-integrated primary production $\langle PP \rangle$ (panel **d**), mixed layer averaged phytoplankton carbon biomass concentration P_{ml} (panel **e**) , depth-integrated phytoplankton carbon biomass $\langle P \rangle$ (panel **f**) , the mixed layer depth H (panel **g**) and the cumulative integral of $\langle PP \rangle$ (panel **h**) . The time axis is rescaled by the onset times of the weak winter accumulation phase (t_1) and the spring bloom (t_2) introducing $\tau = (t - t_1)/(t_2 - t_1)$. $\tau = 0$ corresponds to the initiation of the weak winter accumulation phase and $\tau = 1$ corresponds to the initiation of the spring bloom.

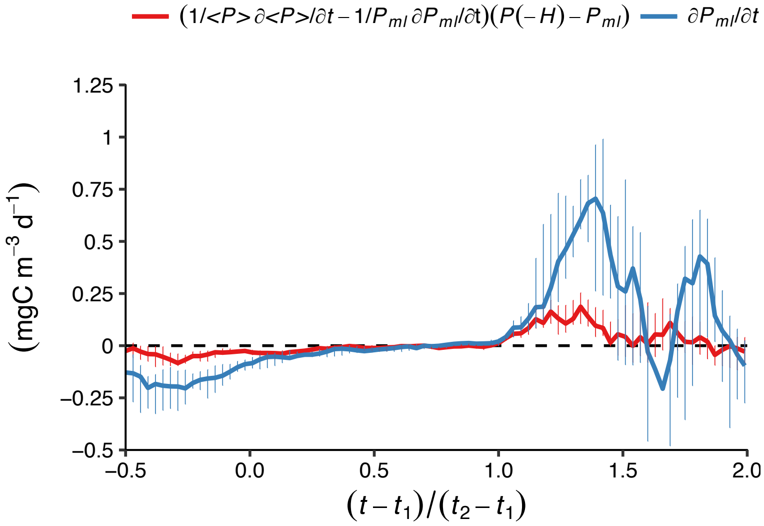

Supplementary Fig. 14. Float estimates of net population growth rates: accuracy of neglecting the detrainment term when the mixed layer shoals. Median (solid thick line) and interquartile range (vertical bars) of $(P(-H) - P_{ml})\left(\frac{1}{\langle P\rangle}\frac{\partial\langle P\rangle}{\partial t} - \frac{1}{P_{ml}}\frac{\partial P_{ml}}{\partial t}\right)$ (red) and $\frac{\partial P_{ml}}{\partial t}$ (blue) over all 12 time series. The time axis is rescaled by the onset times of the weak winter accumulation phase (t_1) and the spring bloom (t_2) introducing $\tau = (t - t_1)/(t_2 - t_1)$. $\tau = 0$ corresponds to the initiation of the weak winter accumulation phase and $\tau = 1$ corresponds to the initiation of the spring bloom.

Supplementary Note 1: Sensitivity of the results to the definition of mixed layer and euphotic layer depths

In our study, the mixed layer depth, H, was computed as the depth at which the potential density change from its value at 10 m, $\Delta\sigma_\theta$, exceeded 0.03 kg m^{-3}, — this value of $\Delta\sigma_\theta$ best tracked the region of weak stratification in our dataset. The euphotic layer depth, H_e, the depth below which the light level is too low to support photosynthesis, was computed as the depth at which the daily-averaged PAR dropped below a threshold level of I_{th} = 0.1 mol photons m^{-2} d^{-1}, corresponding to the lowest light levels at which the temperate diatom *Phaeodactylum tricornutum* has been observed to grow[1]. Other criteria to define mixed layer and euphotic layer depths can be found in the literature and they all have limitations. For example in the study of Boss and Behrenfeld [2] , the threshold light level was set to 0.415 mol photons m^{-2} d^{-1}, and the mixed layer depth was computed as the depth at which the potential density change from its value at 10 m, $\Delta\sigma_\theta$, exceeded 0.125 kg m^{-3}.

To address the sensitivity of our results to the definitions of mixed layer and euphotic layer depths, we plotted the net population growth rates using four different combinations of $\Delta\sigma_\theta$ and I_{th}. Supplementary Figure 15 shows that the time series of r_p are rather similar whether the base of the mixed layer is estimated using $\Delta\sigma_\theta$ = 0.03 kg m^{-3} or $\Delta\sigma_\theta$ = 0.125 kg m^{-3} and the euphotic layer is estimated using I_{th} = 0.1 mol photons m^{-2} d^{-1} or I_{th} = 0.415 mol photons m^{-2} d^{-1}.

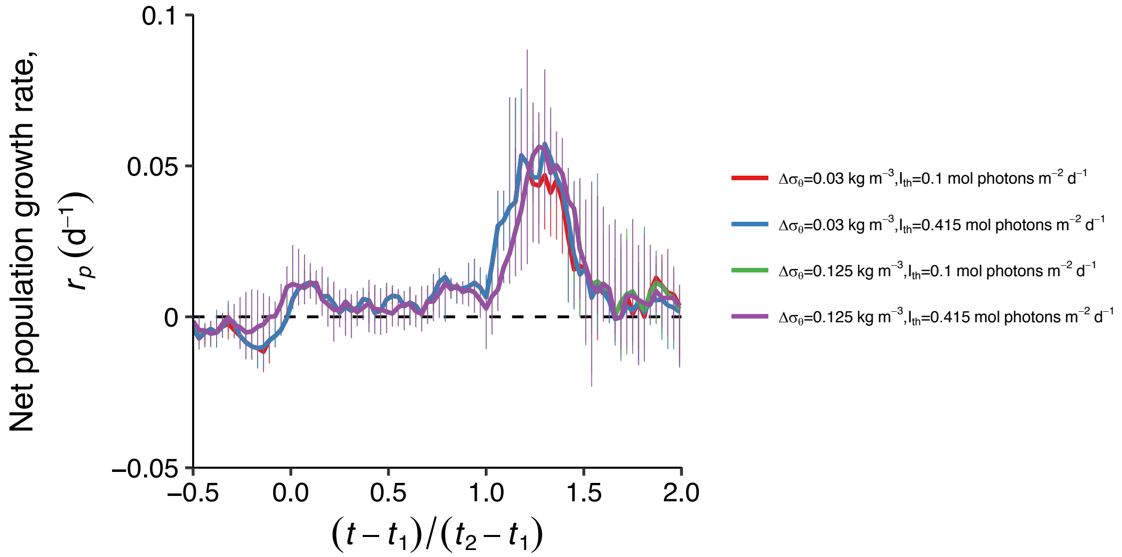

Supplementary Fig. 15 . Median (solid thick line) and interquartile range (vertical bars) of the net population growth rate over all 12 time series. Net population growth rates were computed using 4 different combinations of $\Delta\sigma_\theta$ and I_{th}. The time axis is rescaled by the onset times of the weak winter accumulation phase (t_1) and the spring bloom (t_2) introducing $\tau = (t - t_1)/(t_2 - t_1)$. $\tau = 0$ corresponds to the initiation of the weak winter accumulation phase and $\tau = 1$ corresponds to the initiation of the spring bloom.

Supplementary Note 2: Net population growth rate computed from fluorescence measurements of chlorophyll *a*

Supplementary Figure 16 shows the temporal evolution of b_{bp}-based and chlorophyll-based net population growth rates (r_p). The main findings and conclusions of the present study are quite independent of whether the net population growth rates are estimated from b_{bp} (hence phytoplankton carbon biomass *P)* or chlorophyll *a* fluorescence. Figure 3 in the main text together with the Supplementary Fig. 16 show that both estimates become positive when mixed layers are deepening and phytoplankton division rates are decreasing, and they accelerate when the mixed layer starts shoaling.

There is however an uncertainty in identifying the exact onset of the weak winter accumulation phase. The difference between the two methods is not surprising because temporal changes in properties other than carbon biomass can affect b_{bp} and chlorophyll *a* fluorescence: b_{bp}-phytoplankton carbon biomass relationship changes as the composition and size distribution of the particle assemblage (phytoplankton and non-algal) changes, whereas chlorophyll *a* is also influenced by the intracellular pigmentation and the accurateness of the fluorescence quenching correction. Our assessment of the literature is that b_{bp}-based r_p is a better estimate of the "true" net population growth rate because (1) the ratio of b_{bp} to phytoplankton carbon biomass appears to be constant over a wide range of trophic conditions in the North Atlantic[3], and (2) the fluorescence quenching correction introduces significant uncertainties which are difficult to evaluate.

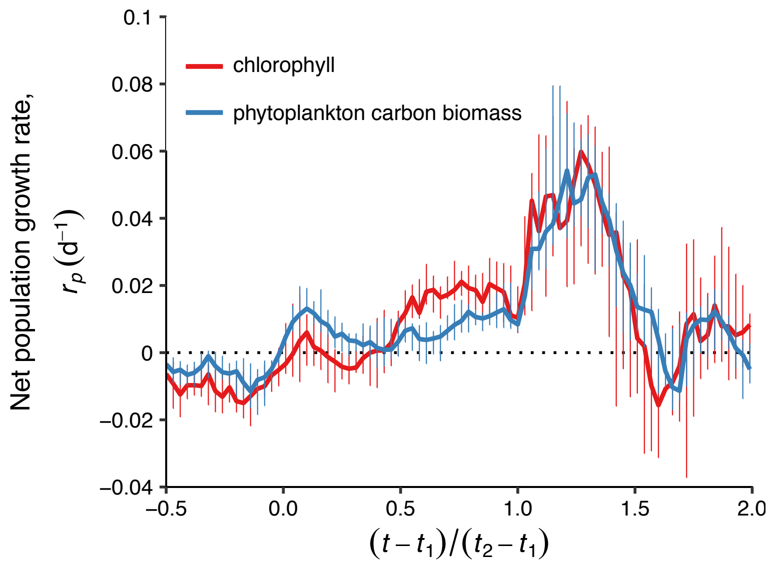

Supplementary Fig. 16. Median (solid thick line) and interquartile range (vertical bars) of the net population growth rate computed from phytoplankton carbon biomass (solid blue line) and computed from fluorescence measurements of chlorophyll *a* (solid red line) over all 12 time series. The time axis is rescaled by the onset times of the weak winter accumulation phase (t_1) and the spring bloom (t_2) introducing $\tau = (t - t_1)/(t_2 - t_1)$. $\tau = 0$ corresponds to the initiation of the weak winter accumulation phase and $\tau = 1$ corresponds to the initiation of the spring bloom.

Supplementary Note 3: Chlorophyll *a* concentrations computed from radiometric measurements

By taking advantage of the link that exist between the diffuse attenuation coefficient for light at 490 nm, *K(490)*, and chlorophyll *a* concentrations in the open ocean [4], float observations of irradiance at 490nm were used to estimate mixed layer-averaged chlorophyll *a* concentrations, Chl_{K490}.

Supplementary Figure 17 shows the comparison between $Chl_{K490,}$ with mixed layer-averaged phytoplankton carbon biomass concentrations derived from b_{bp} measurements, P_{ml} and mixed-layer chlorophyll *a* concentrations derived from fluorescence, Chl_{ml}. The three time series are fairly similar. The three phytoplankton biomass indicators are decreasing at $\tau = 0$ and start accelerating at $\tau = 1$. The major difference occurs in winter: Chl_{K490} is larger than Chl_{ml}. The diffuse attenuation coefficient for light at 490 nm is inversely proportional to the cosine of the zenith angle[5], which is small during winter in the North Atlantic, and results in an increase in *K(490)* unrelated in changes in $Chl_{K490.}$

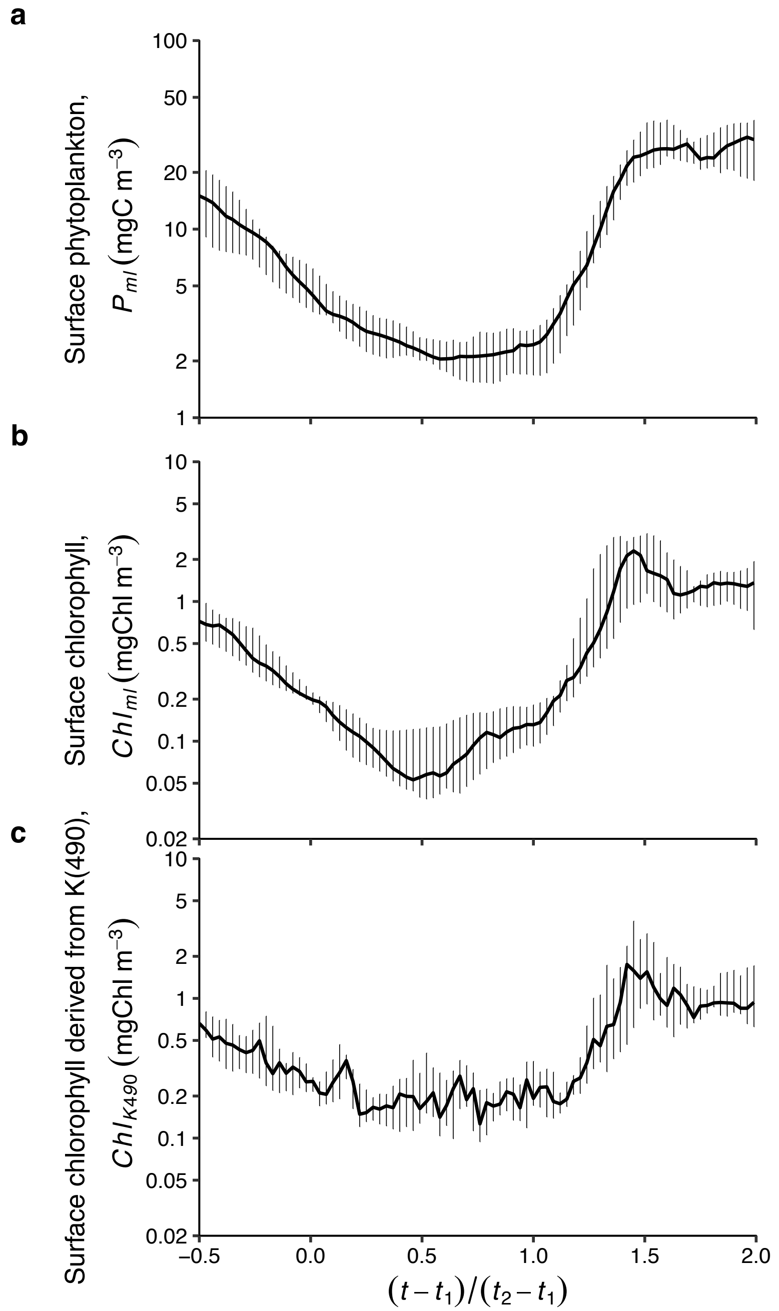

Supplementary Fig. 17 Median (solid thick line) and interquartile range (vertical bars) of (**a**) the mixed layer-averaged phytoplankton carbon biomass concentrations, (**b**) mixed layer-averaged chlorophyll *a* concentrations derived from fluorescence and (**c**) mixed layer-averaged chlorophyll *a* concentrations derived from the diffuse attenuation of light at 490 nm over all 12 time series. The time axis is rescaled by the onset times of the weak

winter accumulation phase (t_1) and the spring bloom (t_2) introducing $\tau = (t - t_1)/(t_2 - t_1)$. $\tau = 0$ corresponds to the initiation of the weak winter accumulation phase and $\tau = 1$ corresponds to the initiation of the spring bloom.

Supplementary Note 4: Low-pass filter timescale

The focus of this work is on the winter-spring phenology of phytoplankton and not on shorter term fluctuations due to transient changes in the environment. Over these two seasons the evolution of phytoplankton is characterized by a slow and continuous population growth in winter and a rapid population growth in spring, the spring bloom. By averaging the time series over the e-folding timescale of the spring bloom, we retain the overall winter-spring phenology of the phytoplankton population (which evolves slower in winter and faster in spring) and at the same time we filter out short term fluctuations. The e-folding timescale of the spring bloom is estimated assuming that mixed layer phytoplankton carbon biomass concentrations (P_{ml}) are exponentially increasing from a winter minimum (P_{min}) to a spring maximum (P_{max}) at a constant rate. The spring bloom timescale is then given by $\frac{t_{max}-t_{min}}{\ln(P_{max})-\ln(P_{min})}$, where t_{min} and t_{max} are the times when the minimum and maximum P_{ml} occur. The spring bloom e-folding timescale estimated from the 12 time series of P_{ml} range from 26 to 35 days. Consequently, we use a running average of 24 days to filter out short term fluctuations in the equally spaced 3-day time series.

Supplementary Note 5: Uncertainties in phytoplankton division and primary production rates estimates.

The main results of the current study are based on the analysis of the temporal variations of the overall phytoplankton division rates (μ_p) and the daily-averaged and depth-integrated primary production (<*PP*>). These rates are estimated using a phytoplankton growth model, as they cannot be directly measured with autonomous platforms. Such models carry substantial uncertainties[6,7] both due to errors in the input variables, *H*, H_e, *MLT*, *iPAR*, *K*, *Chl*, and *P*, and the photosynthesis parameters, $P^B{}_{max}$ and α^B, (Methods Eq. 12). However, we expect these uncertainties to affect primarily the absolute values of phytoplankton division and primary production rates, but not their temporal variations. This is based on the assumption that during winter and spring (the time period of importance in this work), the temporal variability of the input variables is much larger than the errors in the input variables and in the photosynthesis parameters.

To test this hypothesis, we performed a sensitivity analysis of the model by varying the input parameters. We varied each input variable and photosynthesis parameter independently of the other parameters/variables by plus or minus a constant percent value. We varied the photosynthesis parameters within their natural range of variability[7], 10 % for $P^B{}_{max}$ and 25 % for α^B. The relative uncertainties in chlorophyll *a* fluorescence (observations affected by non-photochemical quenching excluded) and b_{bp} measurements estimated from fluorometers and backscattering sensors mounted on BGC-Argo floats is ~10 % and 2% respectively[8]. Assuming an uncertainty of 20 % for the conversion factor that relates C_{phyto} to b_{bp}[9], the average relative uncertainty in our C_{phyto}

estimates is therefore ~20%. The uncertainty in Argo float-based estimates of temperature is ~10% in the subpolar North Atlantic[10]. The uncertainties in *iPAR* and *K* were arbitrarily set to 30 % as no estimates could be found. Finally, the uncertainties in *H*, H_e were set to 25 % and 20%. These values represent the normalized root mean square deviation between mixed layer depths estimated using $\Delta\sigma_\theta$=0.03 kg m^{-3} and $\Delta\sigma_\theta$=0.125 kg m^{-3} and euphotic layer depths estimated using I_{th} = 0.1 mol photons m^{-2} d^{-1} and I_{th} = 0.415 mol photons m^{-2} d^{-1} (see section "Supplementary Note 1").

Results from the sensitivity analysis of the model using as input variables the observations measured by float 6901516 from September 2014 to July 2015 are shown in Supplementary Figs. 18 and 19. The analysis indicates that uncertainties in the input variables and photo-physiological parameters primarily affect the magnitude of μ_p and <*PP*> but much less their temporal evolutions. The same result was found for the 11 other time series considered in the study. We thus conclude that the temporal variations of μ_p and <*PP*> are quite independent of the uncertainties in the input variables and in the photosynthesis parameters and rather reflect temporal variations of the input variables.

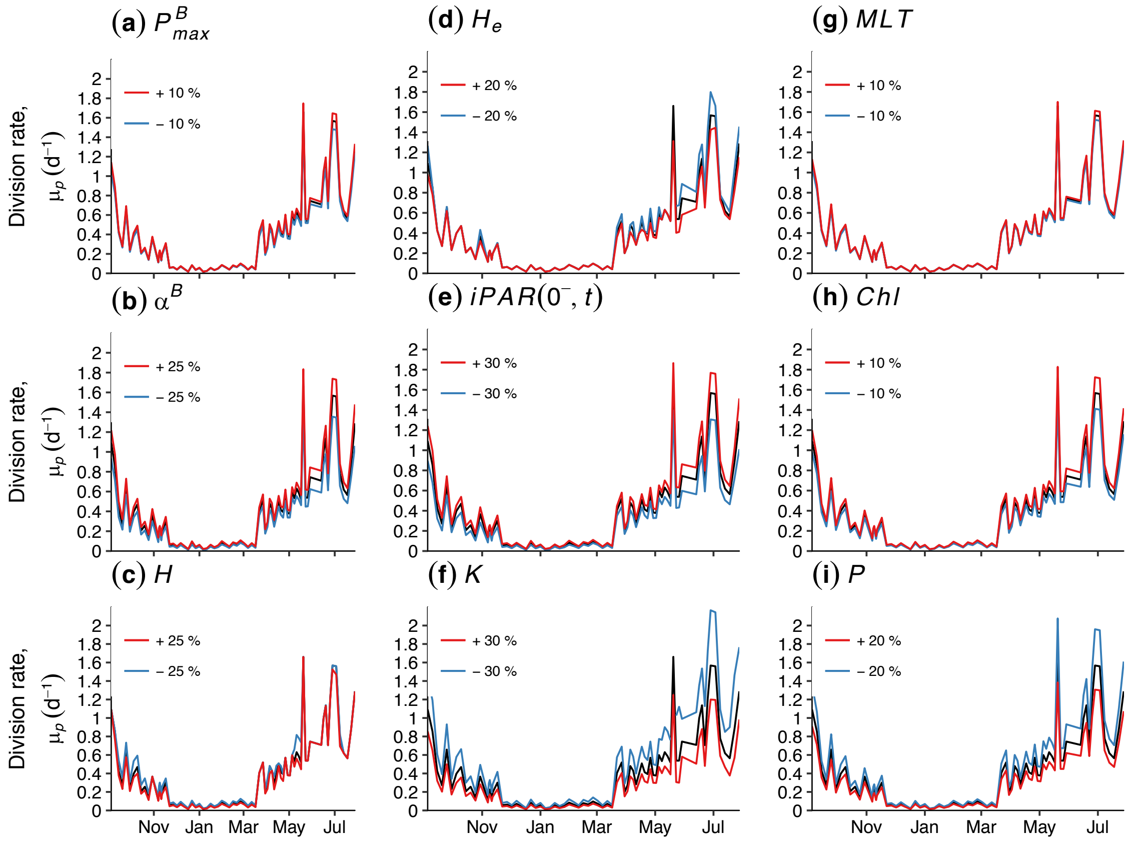

Supplementary Fig. 18. Comparison of time series of μ_p computed using the phytoplankton growth model (black continuous line, as described in the Method Section) with values calculated by varying each input variable (panels **a**-**b**) and photosynthesis parameters (panels **c**-**i**) independently of the other variables/parameters by plus (red continuous line) or minus (blue continuous line) a constant percent value. (**a**) The maximum chlorophyll-specific light saturated photosynthesis, P^B_{max}. (**b**) The chlorophyll-specific initial slope of the photosynthesis-irradiance curve, α^B. (**c**) The mixed layer depth, H (**d**) The euphotic layer depth, H_e. (**e**) The instantaneous photosynthetically available radiation just beneath the sea surface, $iPAR(0^-, t)$. (**f**) The diffuse attenuation coefficient for photosynthetically available radiation, K. (**g**) The mixed layer-averaged potential temperature, MLT. (**h**) Chlorophyll a concentration, Chl. (**i**) Phytoplankton carbon biomass, P.

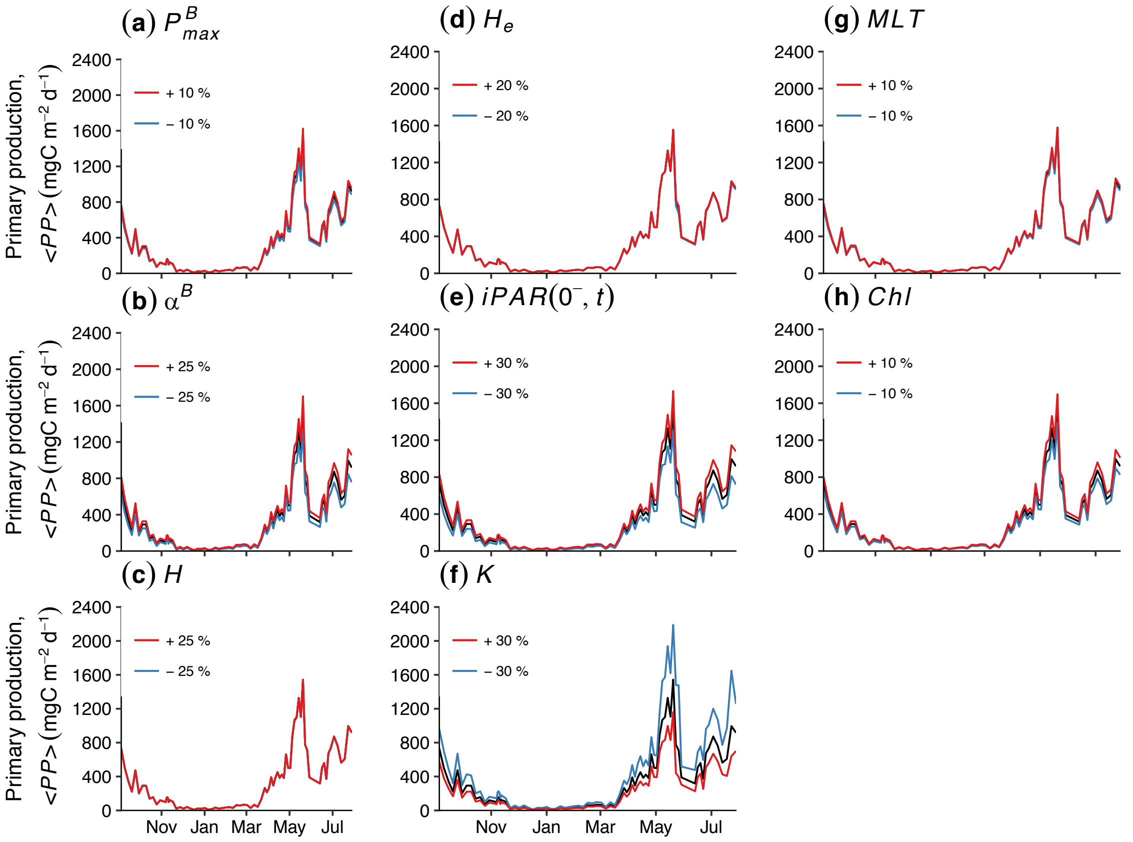

Supplementary Fig. 19. Comparison of time series of <*PP*> computed using the phytoplankton growth model (black continuous line, as described in the Method Section) with values calculated by varying each input variable (panels **a**-**b**) and photosynthesis parameters (panels **c**-**h**) independently of the other variables/parameters by plus (red continuous line) or minus (blue continuous line) a constant percent value. (**a**) The maximum chlorophyll-specific light saturated photosynthesis, P^B_{max}. (**b**) The chlorophyll-specific initial slope of the photosynthesis-irradiance curve, α^B. (**c**) The mixed layer depth, H (**d**) The euphotic layer depth, H_e. (**e**) The instantaneous photosynthetically available radiation just beneath the sea surface, $iPAR(0^-, t)$. (**f**) The diffuse attenuation coefficient for photosynthetically available radiation, K. (**g**) The mixed layer-averaged potential temperature, MLT. (**h**) Chlorophyll a concentration, Chl. Note that the estimates of <*PP*> do not depend on phytoplankton carbon biomass, *P*.

Supplementary References

1. Geider, R., Osborne, B. & Raven, J. Growth, Photosynthesis and Maintenance Metabolic Cost in the Diatom Phaeodactylum-Tricornutum at Very Low Light Levels. *J. Phycol.* **22,** 39–48 (1986).
2. Boss, E. & Behrenfeld, M. In situ evaluation of the initiation of the North Atlantic phytoplankton bloom. *Geophys. Res. Lett.* **37,** L18603 (2010).
3. Graff, J. R. *et al.* Analytical phytoplankton carbon measurements spanning diverse ecosystems. *Deep Sea Res. Part Oceanogr. Res. Pap.* **102,** 16–25 (2015).
4. Morel, A. & Maritorena, S. Bio-optical properties of oceanic waters: A reappraisal. *J. Geophys. Res.-Oceans* **106,** 7163–7180 (2001).
5. Gordon, H. & McCluney, W. Estimation of Depth of Sunlight Penetration in Sea for Remote-Sensing. *Appl. Opt.* **14,** 413–416 (1975).
6. Friedrichs, M. A. M. *et al.* Assessing the uncertainties of model estimates of primary productivity in the tropical Pacific Ocean. *J. Mar. Syst.* **76,** 113–133 (2009).
7. Platt, T. *et al.* Remote Sensing of Primary Production in the Ocean: Promise and Fulfilment [and Discussion]. *Philos. Trans. R. Soc. B Biol. Sci.* **348,** 191–202 (1995).
8. Haëntjens, N. Revisiting Ocean Color algorithms for chlorophyll a and particulate organic carbon in the Southern Ocean using biogeochemical floats. (The University of Maine, 2017).
9. Behrenfeld, M. J., Boss, E., Siegel, D. A. & Shea, D. M. Carbon-based ocean productivity and phytoplankton physiology from space. *Glob. Biogeochem. Cycles* **19,** GB1006 (2005).

10. Hadfield, R. E., Wells, N. C., Josey, S. A. & Hirschi, J. J.-M. On the accuracy of North Atlantic temperature and heat storage fields from Argo. *J. Geophys. Res. Oceans* **112,** C01009 (2007).
